# Advancements in Regenerative Strategies Through the Continuum of Burn Care

**DOI:** 10.3389/fphar.2018.00672

**Published:** 2018-07-09

**Authors:** Randolph Stone II, Shanmugasundaram Natesan, Christine J. Kowalczewski, Lauren H. Mangum, Nicholas E. Clay, Ryan M. Clohessy, Anders H. Carlsson, David H. Tassin, Rodney K. Chan, Julie A. Rizzo, Robert J. Christy

**Affiliations:** ^1^Combat Trauma and Burn Injury Research, US Army Institute of Surgical Research San Antonio, TX, United States; ^2^Extremity Trauma and Regenerative Medicine, US Army Institute of Surgical Research San Antonio, TX, United States; ^3^Dental and Craniofacial Trauma Research, US Army Institute of Surgical Research San Antonio, TX, United States; ^4^Burn Flight Team, US Army Institute of Surgical Research San Antonio, TX, United States

**Keywords:** wound healing, burns, tissue engineering, biomaterials, drug delivery, growth factors, stem cells, regenerative medicine

## Abstract

Burns are caused by several mechanisms including flame, scald, chemical, electrical, and ionizing and non-ionizing radiation. Approximately half a million burn cases are registered annually, of which 40 thousand patients are hospitalized and receive definitive treatment. Burn care is very resource intensive as the treatment regimens and length of hospitalization are substantial. Burn wounds are classified based on depth as superficial (first degree), partial-thickness (second degree), or full-thickness (third degree), which determines the treatment necessary for successful healing. The goal of burn wound care is to fully restore the barrier function of the tissue as quickly as possible while minimizing infection, scarring, and contracture. The aim of this review is to highlight how tissue engineering and regenerative medicine strategies are being used to address the unique challenges of burn wound healing and define the current gaps in care for both partial- and full-thickness burn injuries. This review will present the current standard of care (SOC) and provide information on various treatment options that have been tested pre-clinically or are currently in clinical trials. Due to the complexity of burn wound healing compared to other skin injuries, burn specific treatment regimens must be developed. Recently, tissue engineering and regenerative medicine strategies have been developed to improve skin regeneration that can restore normal skin physiology and limit adverse outcomes, such as infection, delayed re-epithelialization, and scarring. Our emphasis will be centered on how current clinical and pre-clinical research of pharmacological agents, biomaterials, and cellular-based therapies can be applied throughout the continuum of burn care by targeting the stages of wound healing: hemostasis, inflammation, cell proliferation, and matrix remodeling.

## Purpose of the review

Currently, multiple strategies exist for the management of burn wounds depending on both the depth and extent of the burn. Burn wound care strategies aim to modulate the inflammatory response, accelerate re-epithelialization, and improve overall wound healing. Furthermore, combinatorial approaches that incorporate cellular-based therapies, pharmacological agents, and biomaterials are utilized to minimize infection and serve as burn wound coverage adjuncts with the goal of restoration of skin function (i.e., barrier, range of motion, sensation, hair and sweat generation, and pigmentation). This review focuses on how therapies for burn injuries are currently being developed to address the array of issues that occur throughout the continuum of burn care. Specifically, this review investigates treatment modalities for thermal burns that are currently in clinical trials and pre-clinical animal testing. To accomplish this and ultimately illustrate the challenges that remain unmet, it is important to understand the current standard of care (SOC) for burn wound injuries. Next, the United States (US) Food and Drug Administration (FDA) approval process will be described to explain how current products have been approved in order to highlight the challenges that new ideas and technologies will encounter and how this affects the current design of new products. Finally, an analysis of clinical and pre-clinical studies utilizing the latest regenerative therapies will be presented that are addressing the different stages of burn wound healing.

## Burn injuries

### Anatomy of skin

Skin is the human body's largest organ, encompassing ~1.5–2.0 square meters for an average adult. It functions as a defensive barrier against foreign materials, assists in thermoregulation, prevents evaporative loss of fluids, acts as a sensory organ, and plays a role in Vitamin D production. It is composed of three layers: the epidermis, dermis, and hypodermis. The epidermis is the outermost layer, while the dermis is between the epidermis and hypodermis. The papillary dermis (i.e., upper dermal layer) consists of rete ridges, capillaries, and loosely arranged collagen fibers. The reticular dermis (i.e., lower dermal layer) contains blood vessels, nerves, roots of hair follicles, sebaceous and sweat glands, densely packed collagen fibers, and provides nutritional and structural support to the epidermis. The innermost layer is the hypodermis which consists of subcutaneous adipose tissue and associated blood and lymphatic vessels. This layer provides insulation, cushion from traumatic insults, buoyancy to the body, and possesses some endocrine functions (Marks and Miller, 2013; Fenner and Clark, 2016).

### Burn pathophysiology

Even though burn wounds directly affect the skin, severe burns (>20% total body surface area, TBSA) cause a systemic inflammatory response that results in damage throughout the entire body including the immune system, gastrointestinal system, and muscle. This systemic damage is much more pronounced in burn injuries compared to other forms of trauma (Tiwari, 2012). A hallmark indicator of this stress response is an increase in the metabolic rate or hyper-metabolism which can lead to an overall leaner body mass from the increased metabolic demands (Orgill, 2009; Porter et al., 2016). For this review, we only will be focusing on treatments for the primary burn injury. Management of severe burn injuries requires specialized burn centers staffed with burn specialists. Nutrition, pain control, and rehabilitation are important components of burn care, but will not be addressed here. Decades ago, it was understood that burn wounds were unique and healed slower than other traumatic wounds (Monsaingeon and Molimard, 1976). In contrast to excisional wounds, a burn injury occurs with varying degrees of cellular injury, and even viable tissue adjacent to the burn is affected with altered physiology (Monstrey et al., 2008). These physiologic differences translate to slower healing after burn injury compared to excisional injury. For these reasons, animal models with excisional wounds, even those that form hypertrophic scars (HTS), are difficult to extrapolate to burn wounds (Carlsson et al., 2016). For the scope of this review, we will be focusing on research and therapeutics which specifically target and were tested topically on thermal burn wounds.

### Burn incidence and depth

**Figure 1:**
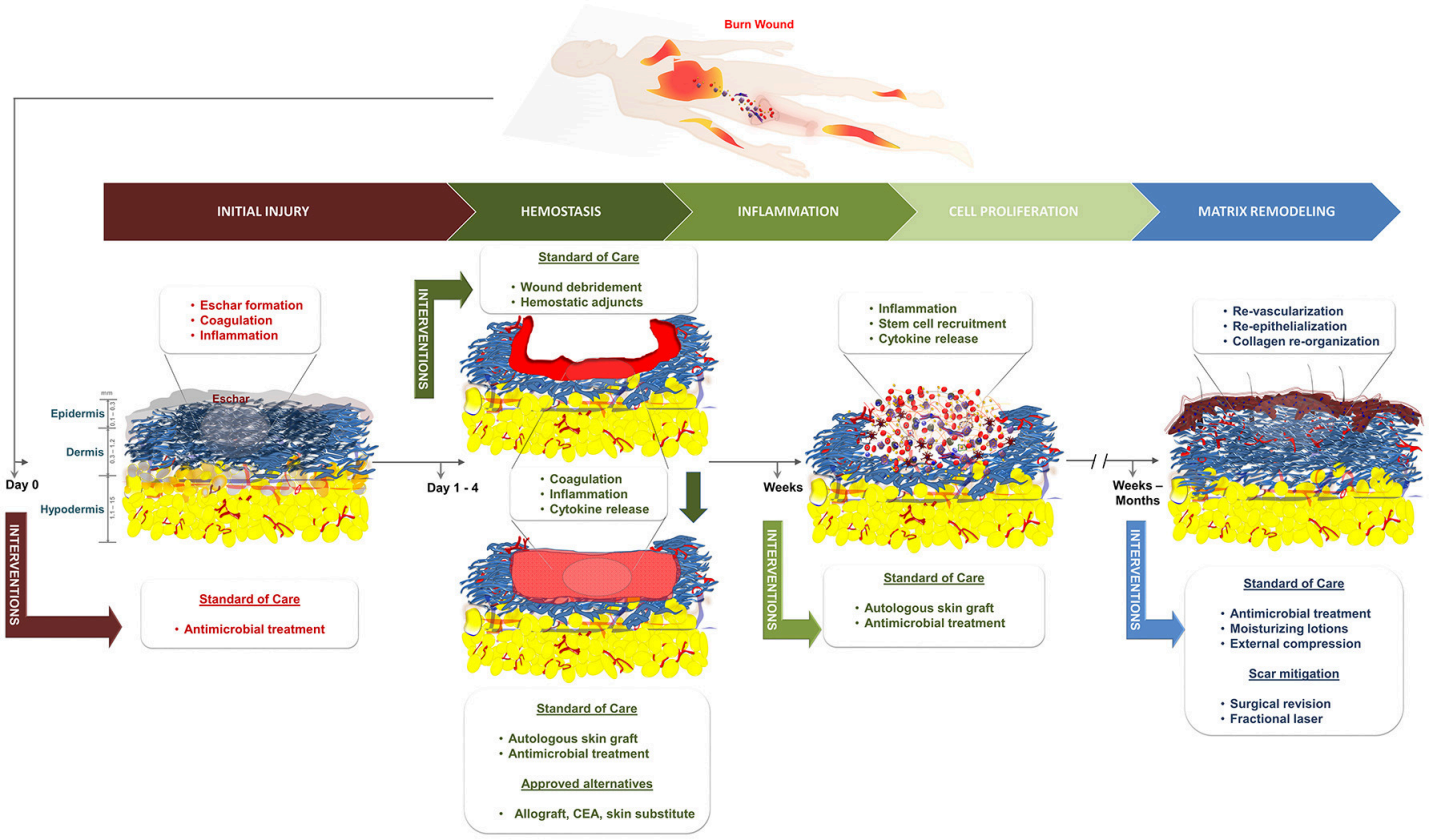
Schematic of the standard of care and the phases of healing for burn wounds. CEA, cultured epithelial autograft.

The annual burn incidence in the United States is ~486,000 according to the National Burn Repository of the American Burn Association. Approximately 3,275 people lose their lives and 40,000 require hospitalization due to burn related injuries (American Burn Association, 2016). A superficial burn involves only the epidermis; a common example is a sunburn which will heal on its own within 7 days by keratinocyte proliferation and differentiation from the basal epithelial cells. Deeper burns can be distinguished based on characteristics including pain (high to none), color (pink/red to white/brown), and capillary refill (brisk to none). Superficial partial-thickness (SPT) burns involve the epidermis and papillary dermis and are very painful to the touch with brisk capillary refill. Deep partial-thickness (DPT) burns involve the reticular dermis including the adnexal structures while full-thickness (FT) burns involve all of the epidermis and dermis and may also affect the subcutaneous adipose tissue, muscle, or even bone. Burn injuries are considered acute wounds that heal via the wound healing cascade (Lazarus et al., 1994; Figure 1). A major clinical challenge is determining the burn depth, which correlates with the amount of time the wound will need to heal. This assessment is extremely important due to the fact that wounds that take longer than 3 weeks to heal on their own have a high risk of forming a HTS (Monstrey et al., 2008). It is estimated that clinical assessment is only accurate ~65% of the time with indeterminate PT burns, where the differentiation between SPT and DPT is difficult (Heimbach et al., 1984; Zuo et al., 2017). A myriad of non-invasive techniques to assess the burn depth have been extensively explored in the clinical and pre-clinical setting. Currently, laser-Doppler imaging, which measures the perfusion or lack thereof in burned tissue, is the only modality that has earned FDA approval for burn assessment (Monstrey et al., 2008).

### Standard of care

#### Infection prevention

After the initial burn injury, infection is the primary cause of death and morbidity, with 51% of deaths attributed to underlying sepsis (Greenhalgh et al., 2007; Krishnan et al., 2013). The wound rapidly becomes colonized by a number of pathogens immediately after injury due to the compromises in the barrier function of the skin. In addition, the bi-phasic immune response, acute hyperinflammation followed by immunosuppression, seen in burns leaves the patient unable to combat the infection. Also, the burn eschar provides an ideal environment that is rich in nutrients (i.e., denatured proteins and lipids) at an ideal temperature for microbial growth (Taneja et al., 2013). To prevent burn wound infection, topical antimicrobial agents have been the mainstay of therapy and include creams [e.g., bacitracin, mupirocin, and silver sulfadiazine (SSD)] and aqueous solutions (e.g., mafenide acetate aka Sulfamylon® and silver nitrate) (Palmieri and Greenhalgh, 2002; Storm-Versloot et al., 2010; Aziz et al., 2012; Heyneman et al., 2016; Afshari et al., 2017; Norman et al., 2017). In order to maintain their efficacy as prophylaxis for infection, these topical agents need to be applied at least daily, which can increase patient pain and interfere with wound healing. Compared to topical agents, silver-based dressings are advantageous as they require less frequent dressing changes due to the sustained release of silver ions to the wound bed. Additionally, clinical studies show that silver-based dressings minimize the incidence of infection, reduce eschar formation, and provide control of wound exudate (Aziz et al., 2012; Wasiak et al., 2013; Marx and Barillo, 2014; Lindberg et al., 2015; Munteanu et al., 2016). However, each topical therapy carries its own risk profile. SSD and silver-based wound dressings have been associated with delayed or incomplete re-epithelialization, generation of discolored scars, limited penetration of the burn eschar, hypersensitivity, neutropenia, and ineffectiveness against some pathogens (Hussain and Ferguson, 2006; Wang et al., 2009a,b). Additionally, silver-based dressings function only when moistened and are relatively expensive; although, these costs are partially mitigated by the need for less frequent dressing applications or changes. Nevertheless, further prospective, randomized control trials (RCT) are needed to determine the optimal wound dressing after burn injury (Storm-Versloot et al., 2010; Jull et al., 2015; Norman et al., 2017).

Due to prolonged hospitalizations after severe burn injury (Sarabahi et al., 2012), there has been a surge of fungal colonization and invasive infections which are linked to high mortality (Norbury et al., 2016; Sharma et al., 2016). Clinical guidelines recommend preventative measures by wound debridement and immediate autografting; otherwise, antifungal drugs such as the echinocandin drug caspofungin (Pappas et al., 2009), voriconazole, nystatin, or amphotericin B (Struck and Gille, 2013; Norbury et al., 2016) may be used. The increasing trend in fungal burn infections signifies an emergence of the next critical obstacle in burn care.

#### Surgical management

Wound management of DPT and FT burn injuries is resource and labor intensive as it often requires multiple surgeries, repeated wound care, and can result in long hospital and intensive care unit stays (Sanchez et al., 2008). Burn wound management costs increase substantially with increasing TBSA primarily due to the length of hospital stay, generally estimated at 1 day per every 1% TBSA burned (Bessey et al., 2014; Kearns et al., 2014; Mathews et al., 2017). Clinically, burn wound management occurs during the acute phase (within days) of burn injury and involves tangential excision of necrotic tissue until punctate bleeding in the wound bed is visible, followed by immediate application of either a permanent autologous skin graft or temporary skin substitute. It is well accepted that early excision and immediate wound coverage help attenuate the inflammatory response of burn injury, decrease risk of infection, and lead to better healing outcomes. Unfortunately, burn wound excision is often accompanied by high amounts of blood loss and hypothermia, both of which limits the amount of tissue that can be excised per operation. Techniques to reduce blood loss include the topical use of thrombin spray, epinephrine soaked gauze, tumescence (subcutaneous infiltration of vasoconstrictors), fibrin sealants, extremity tourniquets, and cautery (Zuo et al., 2017). Complete hemostasis is necessary prior to application of a skin graft in order to prevent hematoma formation which could result in graft failure (Butts et al., 2017).

#### Permanent wound coverage

Autologous split-thickness skin grafts are harvested using a dermatome at a thickness of 0.008”−0.015” and consist of the epidermis and a small portion of the papillary dermis. They are the current SOC for permanent wound coverage for DPT and FT burns. Successful wound coverage with autograft necessitates sufficient donor site availability, which is an issue in larger TBSA burns. Due to the minimal amount of dermis in the autograft, the wounds typically heal with some degree of contraction (Bush and Gertzman, 2016). Meshing the autograft, which cuts slits to expand the skin, is routinely used from 1:1 for smaller burns to 6:1 for large TBSA burns. This process is performed when donor sites are limited in an effort to cover the wound with the minimal amount of required tissue (Finnerty et al., 2016). However, utilizing a higher meshed autograft increases the risk of infection, contraction, and scarring due to a longer time for complete re-epithelialization of the larger interstitial spaces (Finnerty et al., 2016). The hands, neck, and face are areas in which contraction creates dramatic quality of life issues, such as loss of function and poor cosmesis; therefore, unmeshed or sheet skin grafts are typically used to prevent these adverse outcomes. The thickness of the autograft inversely correlates with the amount of contraction that occurs (e.g., a thicker graft results in less contraction) (Carlsson et al., 2016); therefore, a FT graft also may be used in these areas to obtain the best functional and cosmetic outcome. However, utilization of a FT graft generates a secondary FT donor site wound which carries with it increased pain, greater risk of HTS, longer healing time, and its own need for wound closure (Stekelenburg et al., 2016).

#### Temporary wound coverage

Autografts provide the best permanent wound coverage and are always the clinician's first choice when available. However, large TBSA burns may not have autologous skin available due to a lack of donor sites. These cases require temporary wound coverage, such as coverage with fresh or cryopreserved allograft (see Table 7 for available allografts) or the use of a skin substitute until a donor site is ready for re-harvesting (see Table 8 for available substitutes). These temporary biological coverings protect the wound bed from desiccation, heat loss, microbial contamination, and promote the formation of granulation tissue (GT) favorable for autograft placement (Saffle, 2009). Allografts are available through tissue banks regulated by the American Association of Tissue Banks (AATB). Fresh allografts possess viable cells; however, the donor epidermis is not immune privileged and will ultimately be rejected due to the presence of class II antigens on the surface of the Langerhans cells. Since the dermis consists of mostly inert collagen, it can be incorporated into the GT of the wound bed, ultimately supporting a future autograft (Voigt et al., 2018). A wide variety of skin substitute products harvested either from cadaveric humans (allograft) or animals (xenograft) are available and consist of dermis and possibly even epidermis with the intent of replacing “like with like” (Tables 7, 8). Other than Epicel®, these substitutes are not autologous and do not provide permanent coverage of the wound but instead can provide temporary coverage with a benefit of augmenting the regeneration of the missing dermis. A few of these devices consist of an outer silicone layer to mimic a few of the epidermal functions such as preventing desiccation and bacterial contamination of the wound bed. A recent survey of 500 burn care specialists worldwide found that 51 and 28% frequently use allografts and xenografts, respectively, for temporary coverage of burn wounds with the intent of establishing an optimal wound bed to support wound closure with a meshed autograft (Wurzer et al., 2016). This two-stage method is commonly employed in which tangential excision and temporary coverage is performed during the first surgery and after a certain amount of time and improvement in the patient's condition, a skin graft is applied during a subsequent operation. The survey revealed that 61% of respondents use biological or synthetic materials in clinical practice but agreed that no ideal skin substitute exists that replaces all the characteristics of skin (Wurzer et al., 2016).

#### Hypertrophic scar (HTS) prevention

As stated above, if a wound has not healed in 3 weeks there is a high risk of developing HTS. Other risk factors of HTS formation include age (children), darker skin color (pigmentation), female gender, facial or neck injuries, and severity of injury (%TBSA and depth). Several studies report that from 32 to 72% of all burns result in HTS formation (Bombaro et al., 2003; Lawrence et al., 2012; Finnerty et al., 2016). Currently, no consensus exists as to the best method to prevent HTS formation. Commonly used techniques to mitigate HTS formation include massage therapy, moisturizers, pressure garments, silicone gel sheets, and exercise with varying results (Anthonissen et al., 2016). These methods are widely available, inexpensive, and low-risk but with controversial efficacy, as even large comprehensive reviews find only low quality evidence supporting their use in some cases (O'Brien and Jones, 2013). Nevertheless, these easy-to-use options have few adverse effects and remain a part of common clinical practice.

#### Hypertrophic scar (HTS) mitigation

Scar revision surgery remains the definitive method for managing HTS, particularly those with associated contractures. Surgeons employ many techniques to release the contracture with a complexity ranging from simple incision with a blade across the scar and application of skin substitute and/or autograft to local perforator flaps to more complex free flap reconstructions that may involve tissue expanders (Hudson and Renshaw, 2006; Hayashida and Akita, 2017). Other first-line treatments are also available that are less invasive. Intralesional steroid injection, for instance, inhibits fibroblast activity and alters transforming growth factor beta's (TGF-β_1_and TGF-β_2_*)* expression (Tziotzios et al., 2012). Similarly, intralesional 5-fluorouracil injections inhibit collagen production and fibroblast proliferation (de Waard et al., 1998), limiting the severity of HTS. These therapies are generally well tolerated with minimal side effects such as hypopigmentation or dermal atrophy. Cryotherapy is a safe office-based procedure often used in conjunction with or as a second-line treatment after steroid injection. Liquid nitrogen is carefully sprayed onto a scar, freezing and lysing underlying cells to alter fibrotic tissue, fibroblast activity, and collagen synthesis (Dalkowski et al., 2003), thereby reducing scar thickness. Lastly, clinicians are beginning to use a variety of medical lasers to treat HTS, which will be discussed later in section HTS Mitigation of the review. Many of these treatments are often combined over the course of a patient's care with synergistic results. Unfortunately, none of the available treatments have resulted in complete abatement of HTS.

## FDA regulations

Navigating the FDA system is complex and not a focus of this review but a basic understanding of the approval process is warranted since it impacts the development of future products. The FDA regulates most drugs, biologics, medical devices, and human cells and tissue products (HCT/P) by one of the following mechanisms with the goal of evaluating the safety and efficacy of a new product: (1) Investigational New Drug (IND) and New Drug Application (NDA) or Biological License Application (BLA), (2) 510(k) Submissions, (3) Investigational Device Exemption (IDE) and Premarket Approvals (PMA), (4) Humanitarian Device Exemptions (HDE), and (5) HCT/P. Table 1 explains each category, lists examples, outlines the steps during the approval process, and indicates if a clinical trial is required.

**Table 1:** FDA approval mechanisms.

**Designations**	**FDA definition**	**Examples**	**Approval process**
Drug	Recognized by an official pharmacopeia or formulary Intended for use in the diagnosis, cure, mitigation, treatment, or prevention of disease Intended to affect the structure or any function of the body Intended for use as a component of a medicine but not a device	Prescription drugs:Brand name Generic Over the counter	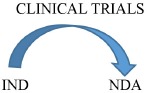
Biologic	Vaccines Blood and blood components Allergenics Somatic cells Gene therapy Tissues Recombinant therapeutic proteins	Self-explanatory	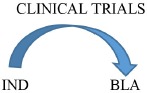
Class I medical device	Devices not intended to: Support or sustain human life Prevent impairment to human life Present a potential unreasonable risk of illness or injury	Tongue depressors; Gloves; Adhesive bandages	Regulated under “General Controls” (ensures safety, effectiveness, and adherence to labeling and good manufacturing practices)
Class II medical device	Devices in which general controls are insufficient in providing reasonable assurances about the safety and effectiveness Moderate to high risk to human life	Automated Blood Analyzer; Acellular dermal matrices; Glucose monitoring systems	Regulated under “General Controls” plus “Special Controls” (performance standards, post-market surveillance, patient registries, special labeling), and 510 (k) “cleared” based on “substantial equivalence” to a “predicate” (current legally marketed) device
Class III medical device	Devices that may support or sustain human life, or prevent impairment of human health, or that may present a potential unreasonable risk of illness or injury High risk to human life	Pace Makers; Heart pumps and valves; Ceramic Hip; Implantable spinal cathodes	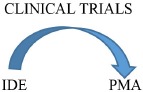
Humanitarian device exemption (HDE)	Medical devices intended for conditions or diseases that affect fewer than 8,000 individuals annually	Deep brain stimulation system; Microsphere radiation treatment for hepatocellular carcinoma; EpiCel® cultured epithelial autografts	Application is similar to PMA but with conditions: No current device on the market for intended disease Exempt from clinical efficacy requirement Can only be used under an IRB Profit and annual distribution limits
Human cellular tissue products (HCT/P)	Human cells or tissue that: “Minimally manipulated” (processed and not significantly changed in structure from the natural material) Intended for “homologous use” (replacing, repairing, or regenerating like tissue)	Donor organs such as skin commonly referred to as “Allograft” (donated from another human)	Exempt, FDA regulates the AATB approved facilities

*AATB, American association of tissue banks; BLA, Biologic license application; IDE, Investigation device exemption; IND, Investigational new drug; NDA, New drug application; PMA, Pre-market approval*.

Substantial Equivalence is a determination made by the FDA to grant 510 (k) clearance.

Is the predicate device legally marketed?

Do the devices have the same intended use?

Do the devices have the same technological characteristics?

If not, are there new questions of safety and effectiveness?

Does the performance data demonstrate substantial equivalence?

Drugs and biologics (i.e., stem cell therapies) take the longest to acquire approval that begins with pre-clinical animal studies followed by three phases of clinical trials, which are required components for an NDA or BLA submission to the FDA. This entire process can take 7–15 years and the latest estimated costs are ~$1B (Ciociola et al., 2014; Hung et al., 2017). Medical devices have 3 classes (I, II, and III) that correspond to increasing levels of risk for human use. Class I devices are regulated under general controls and are generally accepted as no risk to human life. Class II devices (i.e., some dressings and skin substitutes/acellular dermal matrices) require a 510(k) submission which is a review by the FDA to determine “substantial equivalence (SE).” This simply states that the new device has similar characteristics and intended use as a legally marketed device and is “cleared” for commercial distribution. It is noteworthy that most medical devices are “cleared” in this manner, which is not an actual “approval” of the device by the FDA and often does not require any human clinical data, with ~3,000–4,000 devices 510 (k) “cleared” annually (www.FDA.gov). Class III devices support or sustain human life thus must obtain a PMA after progressing through clinical trials to establish the device's safety. The HDE designation is for specialty diseases that affect a limited number of individuals (8,000) every year. The exemption is accompanied by very strict guidelines with the intent on getting these products to market in a quicker fashion. HCT/P products (i.e., allografts) are available through tissue banks much like blood through blood banks. The tissue banks must adhere to standards set forth by the FDA and the products must be screened and verified that no viral, such as human HIV and hepatitis, or bacterial contaminants are present (FDA, 2017, 2018a,b,c). In the following sections, we have listed products used in burn care that have been granted FDA approval via one of these mechanisms. The intention is to bring to light the plethora of available products with either a 510 (k) or HCT/P approval (Tables 5, 7, 8) but in some cases no thorough clinical evaluation has been performed showing true efficacy.

## Wound healing cascade

When skin is injured as a result of trauma, surgery, or burn, it is considered an acute wound. Wound healing is an orchestrated sequence of events involving chemical signals, extracellular matrix (ECM) molecules, and a wide range of cell types. Acute wound healing follows a complex, overlapping cascade of events consisting of hemostasis, inflammation, cell proliferation, and matrix remodeling of the wound site (Lazarus et al., 1994). Table 2 lists the endogenous cells that are involved in this healing process and indicates what they secrete, the cells that those cytokines and growth factors target, and the subsequent cellular response. Each of these stages of wound healing are potential targets for tissue engineering and regenerative medicine (TERM) and regenerative pharmacological strategies.

**Table 2:** Cellular responses throughout wound healing.

**Cell types**	**Key secretome**	**Target**	**Response**
**HEMOSTASIS**			
Red blood cells 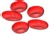	Hemoglobin, oxygen, ATP, nitric oxide	Binds to endothelial cells, platelets, matrix proteins, self-adhesion	Coagulation/clot formation, free radicals generated breaks down bacterial cell wall
Platelets 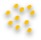	Growth factors (FGF, TGF-α/β, PDGF, IGF, VEGF), vWF, fibrinogen, fibronectin, platelet factor 4, ADP, ATP, calcium thromboxane, thromboplastin	Keratinocytes, endothelial cells, macrophages, fibroblasts	Clot formation, keratinocyte, endothelial and fibroblast cell migration, macrophage activation, and provisional matrix production
**INFLAMMATION**			
Neutrophils 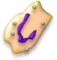	TNF-α, IL-6, IL-8, M IP-1α, IL-1β, CXCL2, G-CSF, NF-κB, opsonins, IgG, myeloperoxidase, elastase	Pathogens, lymphocytes, macrophages, dendritic cells, endothelial, and epithelial cells	Phagocytosis, degranulation, initiate inflammation, homeostasis
Basophils 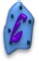	Heparin, histamine, leukotrienes, IL-4, IL-13	T lymphocytes (T_H_ 2 cells), platelets	Vasodilator, innate immune response
Eosinophils 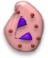	Eosinophil peroxidase, PGE-2, platelet-activating factor, various interleukins and chemokines, IDO	Neutrophils, macrophages, platelets	Initiates early immune response
Dendritic cells 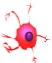	TNF-α, IL-1β, INF-γ, IL-12	Pathogenic microbes and viruses; pathogen derived nucleic acid; Interacts and activates T-cells	Pathogen recognition, activation of T-cells, inhibits bacterial and viral replication. Induces early inflammatory response and re-epithelialization of wound
Langerhans cells 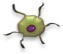	CLA, MHC-I and II molecules	Pathogens, CD8+ T-cells, initiate follicular T_H_ cells, B-cell activation	Epidermal homeostasis, direct keratinocyte proliferation and differentiation
Natural killer cells 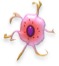	INF-γ, GM-CSF	Infected cells (MHC-I), neutrophils	Cytotoxic innate immunity
**T-cells** Helper T cells 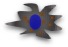	INF-γ, IL-5, IL-10, IL-13, IL-4, IL-2, TNF-α	B-cells, cytotoxic T-cells, CD-40 expressing keratinocytes, fibroblasts, platelets, macrophages	Antibody-driven adaptive immunity
Regulatory T cells 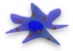	TGF-β, IL-10, IL-2, granzyme, IDO	B-cells, cytotoxic T-cells, macrophages	Antibody-driven adaptive immunity, attenuates INF-γ production and pro-inflammatory macrophage accumulation
Cytotoxic T cells 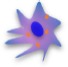	Perforins, granzymes, granulysins, IL-10	MHC-I and II presenting cells	Destroys virus infected cells, necrotic cells, and cell debris
Natural killer T cells 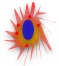	INF-γ, GM-CSF, IL-2, IL-13, IL-17, TNF-α	Neutrophils, macrophages	Attenuates neutrophil response, regulates TGF-β and collagen production
Dendritic epidermal T cells 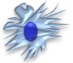	FGF-7, KGF-1, IGF-1, IL-17	Keratinocytes, macrophages	Keratinocyte proliferation, hyaluronan synthesis, enhances antimicrobial function of NK cells
B-Cells 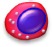	Secretion of antibodies: Immunoglobulin (IgM, IgG, IgE, IgA, IgD)	T_H_2 cells, bacterial and viral antigens	Inactivate toxins, opsonize bacteria, flag pathogens for destruction
M1 Macrophages 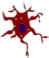	ROS and nitrogen intermediates; pro-inflammatory cytokines (IL-1β, TNF-α, IL-6); chemokines- CXCL9 and CXCL10	Microbes, damaged/necrotic cells, activated lymphocytes- T_H_1 cells, PGE2, PGD2	Potentiates inflammation, phagocytosis, clearance of cellular debris, production of pro-inflammatory mediators
M2 Macrophages 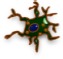	Anti-inflammatory cytokines (IL-4, IL-13, IL-21, IL-10); chemokines (CCL17, CCL22 and CCL24); TGF-β, glucocorticoids, prostaglandins, lipid mediators	Polarized T_H_2 cells, M1 macrophages	Suppress inflammation, efferocytosis, tissue repair-angiogenesis, matrix production
Mast cells 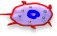	Histamine, serine protease, heparin, chondroitin sulfate, TNF-β, IL-3, GM-CSF, IL-5, IL-6, IL-8, MIP-1β	Smooth muscle cells, endothelial cells, nerve endings, and mucous secretion	Smooth muscle cell contraction, erythema, edema, leukocyte influx
**CELL PROLIFERATION**			
Stem cells (SC) of hematopeitic origin [hematopoetic cells (HSC), endothelial progenitor cells (EPC), very small embryonic like SCs (VSELs)] 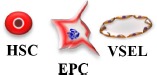	Multiple cytokines and growth factors	Endothelial cells, immune cells (neutrophils, macrophages) fibroblasts, endothelial cells	Promote inflammation and coagulation, vasculogenesis
Stem cells of epidermal origin [basal epithelial (bEpi), follicular (FSC), eccrine gland, dermal papilla (DP), bulge cells (bc)] 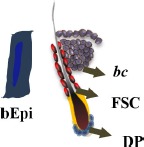	Cytokeratins, growth factors (EGF, TGF-β, VEGF, IGF), basement membrane proteins	Epithelial cells, hair follicles, dermal cells (fibroblasts, endothelial cells, smooth muscle cells), immune cells (dendritic cells, neutrophils, macrophages)	Generation of epithelial cells (keratinocytes and melanocytes), hair follicles, and sweat glands. Promote re-epithelialization
BMSCs and ASCs 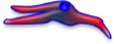	Anti-inflammatory cytokines (TSG-6, PGE2), growth factors (VEGF, CTGF, TGF-β, IGF, bFGF,SDF-1, angiopoeitin, and many others)	Macrophages, lymphocytes, fibroblasts, keratinocytes, adipocytes, endothelial cells, smooth muscle cells	Anti-inflammatory, promote vascularization, re-epithelialization, collagen production, reduces fibrosis and scar formation
Keratinocytes 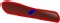	Cytokeratins, membrane proteins (collagen IV,VII, laminin V, perlecan), growth factors (MSF, NGF, VEGF, GM-CSF), cytokines (TNF-α, IL-1-α, β)	Immune cells (neutrophils, macrophages), fibroblasts, melanocytes, bulge cells, endothelial cells	Formation of epithelium, restore barrier function, involve in follicle and sweat gland generation, interacts with fibroblasts and endothelial cells to promote remodeling and angiogenesis
Melanocytes 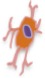	Melanin	Keratinocytes	Barrier function (mainly pigmentation and prevention of uv damage to the skin)
Endothelial cells 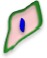	Growth factors (VEGF, TGF-β, FGF, angiopoeitin), ECM proteins (integrins, fibronectin, involucrin)	Multiple cell type (immune cells, fibroblasts, adipocytes, epithelial cells)	Angiogenesis, blood vessel stabilization, origination of stem cells
Fibroblasts (papillary, reticular) 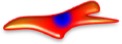	Collagen (multiple types), fibrillin, elastin, enascins, MMPs, TIMPs, glycosaminoglycans (GAGs) and proteoglycans, growth factors (FGF, TGF-β, KGF, GM-CSF)	Multiple cell types (Endothelial cells, epithelial cells, smooth muscle cells, immune cells, adipocytes)	Promotes connective tissue formation, dermal remodeling, interacts with epithelial cells during re-epithelialization
Pericytes (Peri), smooth muscle cells (SMC) 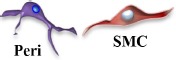	ECM proteins such as actin, integrin, elastin; growth factors (PDGF-β, TGF-β), α-SMA	Endothelial cells, smooth muscle cells, immune cells, adipocytes, fibroblasts	Blood vessel stabilization, immune response, remodeling
Adipocytes (Adipo) (subcutaneous) 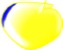	Adipokines (adiponectin, leptin, resistin)	Endothelial cells, smooth muscle cells, pericytes, macrophages, hair follicles, sweat glands	Glucose metabolism, inflammation, influence dermal reorganization, homeostasis, lipid metabolism, angiogenesis

### TERM and regenerative pharmacology

As defined by the National Institute of Health, tissue engineered (TE) refers to “the practice of combining scaffolds, cells, and biologically active molecules into functional tissue….that restore, maintain, or improve damaged tissue or whole organs” (National Institute of Health, 2018). As with other target tissue, TE scaffolds for skin substitutes aim to be biomimetic through the use of native ECM proteins (e.g. collagen, elastin, hyaluronan, fibrin, fibronectin, and chondroitin sulfate) and cells (e.g. keratinocytes and fibroblasts). Regenerative pharmacology is “the application of pharmacological sciences to accelerate, optimize, and characterize (either *in vitro* or *in vivo*) the development, maturation, and function of bioengineered and regenerating tissues” (Christ et al., 2013). It can be used to accelerate wound healing through the delivery of pro-regenerative molecules such as immunomodulators, growth factors, gene therapy, and cell secretomes that can be delivered alone or by a TE construct. Together, TE and regenerative pharmacology fall under the broader umbrella of regenerative medicine, with the goal in which “the body uses its own systems, or sometimes help with foreign biological material to recreate cells and rebuild tissues and organs” (National Institute of Health, 2018). The following sections will indicate how current TERM techniques are being utilized throughout the phases of wound healing.

## Hemostasis

Hemostasis is achieved by platelet accumulation at the site of injury and formation of a fibrin laden blood clot. Growth factors are released from the platelets after thrombin induced degranulation: platelet-derived growth factor (PDGF-α/β), TGF-α/β, and epidermal growth factor (EGF), which are trapped in the blood clot and recruit other cell types for wound repair (Werner and Grose, 2003). During burn surgery, significant amounts of blood loss occur during debridement and excision, estimated at ~200 ml/% TBSA that is tangentially excised (Allorto et al., 2015; Zuo et al., 2017). This presents a significant challenge for large TBSA wounds requiring debridement. For instance, a 50% TBSA patient could lose an estimated 5–10 liters of blood during surgery, thereby exceeding the blood volume of an adult and requiring replacement by transfusion (Zuo et al., 2017). The resulting massive transfusions can lead to a variety of complications such as hemorrhagic shock, infection, acute lung injury, multi-organ dysfunction, and even an increase in mortality (Sterling and Heimbach, 2011).

### Hemostatic adjuncts

A variety of topical hemostatic adjuncts are FDA approved to limit the amount of intraoperative blood loss that occurs during the excision surgery (Table 3) and have been recently reviewed (Sterling and Heimbach, 2011; Shander et al., 2014). In this section, therapies currently in clinical trials or that have recently completed a clinical trial will be discussed (Table 4).

**Table 3:** Hemostatic adjuncts FDA approved for hemostasis.

**Product, company**	**Indication**	**Composition**	**Format**	**FDA approval**
Epinephrine	As a topical hemostatic, solution concentrations of 0.002–0.1% have been sprayed or applied with cotton or gauze to the skin, mucous membranes, or other tissues	Adrenaline (Epinephrine)	FD powder and solution	NDA
Gelfoam®, Pfizer, Inc.	As an adjunct to hemostasis in patients undergoing surgery when control of bleeding by conventional surgical techniques is ineffective or impractical	Gelatin prepared from purified porcine skin	Absorbable gelatin compressed sponge in sheets	PMA
Surgicel®, Ethicon, Inc.	Same as above	Oxidized regenerated cellulose	Knitted fabric strips and sheets	PMA
Thrombin JMI®, Pfizer, Inc.	Same as above	Bovine thrombin	Kit contains FD thrombin, sterile saline, and spray applicator	BLA
Evithrom®, Ethicon, Inc.	Same as above	Human plasma-derived thrombin	Frozen solution	BLA
Recothrom®, ZymoGenetics, Inc.	Same as above	Human recombinant thrombin	FD powder	BLA
Evicel®, Ethicon, Inc.	Same as above	Human plasma-derived fibrinogen and thrombin	One frozen vial of each solution and spray applicator	BLA
Tisseal®, Baxter Healthcare Corp.	Same as above	Human plasma-derived fibrinogen protein concentrate and thrombin	(1) Kit with vials of FD components with reconstitution solutions (2) Pre-filled dual chambered syringe stored frozen	BLA

*BLA, Biological License Application; FD, Freeze-Dried; NDA, New Drug Application; PMA, Pre-Market Approval*.

**Table 4:** Clinical trials for hemostatic adjuncts.

**Clinical trial #**	**Clinical trial title**	**Intervention**	**Characteristics**			
			**Enrollment**	**Model**	**Allocation**	**Phase**
NCT01731444	Phenylephrine tumescence for hemostasis in surgery for burn injury	Drug: Phenylephrine	24	PA	R	1
NCT02012569	Determine the haemostatic efficacy of TT-173, reducing the bleeding time in the donor site of skin grafting (EHTIC)	Drug: TT-173 Drug: Placebo	78	PA	R	2
NCT02148705	A Study to evaluate the efficacy and safety of NexoBrid™ in subjects with thermal burns	Drug: NexoBrid™Procedure: SOCDrug: Gel Vehicle	175	PA	R	3
NCT00371215	Study of recombinant human thrombin for bleeding during autologous skin grafting	Biological: rThrombin	72	SGA	–	2
NCT00859547	Safety and immunogenicity study of recombinant thrombin (rThrombin) in pediatric participants	Biological: rThrombin, 1,000 IU/mL	30	SGA	–	4
NCT00181974	Efficacy of a fibrin sealant in burn surgery	Drug: Tisseel® Fibrin Sealant	25	PA	NR	–
NCT01843686	Using autologous platelet rich plasma (PRP) gel to treat deep 2nd and 3rd degree burns	Device: Magellan®Other: Placebo Saline Gel and SOC	42	PA	R	1

“*–”, study did not mention; SOC, Standard of Care; Model: PA, Parallel Assignment (Therapy vs. SOC); SGA, Single Group Assignment; Allocation: R, Randomized; NR, Non-Randomized*.

Epinephrine, a non-selective agonist of adrenergic receptors which activates α1, α2, β1, and β2 receptors, is part of the SOC and applied as a dilute solution in epi-soaked gauze but also infiltrated under the burn and donor site during the tumescent process. Phenylephrine, a selective agonist of adrenergic receptors which only activates α1 receptor, is being investigated as an alternative to epinephrine as a tumescent solution on the hypothesis of equal efficacy without the systemic side effects due to a lack of α2 and β-adrenergic activity. A recent phase 0 concentration finding study was completed that found vasoconstriction was achieved at a concentration of 5 ug/ml on donor sites in 6 burn patients (Mitchell et al., 2011). A phase 1 RCT is currently underway testing phenylephrine instead of epinephrine for tumescent infiltration of the injured site to decrease blood loss during tangential excision.

A new hemostatic hemafiber dressing, NuStat®, was recently cleared by the FDA that consists of a mixture of bamboo cellulose and continuous filament silica. This unique combination promotes hemostasis chemically by activating the coagulation cascade but also mechanically by compression. In a single institution RCT of burn patients requiring tangential excision, NuStat® was compared to the SOC administration of thrombin and epinephrine-soaked non-adherent dressings. Each patient was their own control with both therapies applied on roughly half of the burn and donor site. No statistically significant differences were observed in the amount of blood loss from either site indicating comparative efficacy to the SOC. Benefits of NuStat® reported were lower cost and ease of application due to no reagent preparation vs. the SOC (Butts et al., 2017).

TT-173 (Thrombotargets, Spain) is a new hemostatic agent that has been developed to modulate the coagulation pathway to induce clotting. It consists of a lipid microvesicle with a modified version of recombinant human tissue factor that is lyophilized and applied as a spray. A phase 2 RCT of 78 patients was recently completed evaluating this product's ability to reduce donor site bleeding duration. TT-173 was shown to stop bleeding faster than placebo. No adverse events were observed and donor sites healed as expected. Other benefits of TT-173 reported were reduced cost compared to fibrin sealants, ease of manufacturing, and no human or animal components which decreases the risk of pathogen transmission (Rojas et al., 2017).

Enzymatic debridement is an alternative debridement method that digests the proteins present in the necrotic tissue of the wound bed. NexoBrid® consists of a group of Bromelain enzymes that are extracted from the fruit and stems of pineapples. This product is currently approved in Europe and has been reported to selectively digest the necrotic tissue and work in as little as 4 h after application. Interestingly and more relevant to this section of the review, an additional benefit is a decrease in intraoperative blood loss, with reports of higher hemoglobin and hematocrit levels in patients treated with NexoBrid® vs. SOC (Rosenberg et al., 2015). A recent European consensus was published that had unanimous responses from surveyed clinicians stating enzymatic debridement with NexoBrid® reduced blood loss compared to SOC (Hirche et al., 2017). Seven clinical trials testing the safety and efficacy of this product have been completed with a US based multicenter RCT currently recruiting burn patients to demonstrate complete eschar removal and reduction in patients' surgical burden and its related blood loss as compared to SOC without long term cosmetic and functional issues (Rosenberg et al., 2014, 2015).

Recent clinical research evaluated the use of rThrombin (already FDA approved, ZymoGenetics, Inc.) as a plasma-free alternative produced from mammalian cells for use during burn surgeries. The initial study demonstrated efficacy in achieving hemostasis with 91.5% of patients attaining it in 20 min and safety with only 1.6% of the patients developing antibodies to the rThrombin (Greenhalgh et al., 2009). In a follow up study in 30 pediatric burn patients, topical rThrombin was applied as a hemostatic agent on day 1 and demonstrated no anti-rThrombin antibody production at day 29 (Foster et al., 2011).

Given the fact that these potential therapies are considered drugs or biologics, there is a long regulatory approval required before these newer TERM strategies can be implemented as SOC.

## Inflammation

Inflammation, the second phase of wound healing, follows a general pattern of cellular infiltration following injury. Polymorphonuclear leukocytes (PMNs) (Table 2), a category of white blood cells which includes basophils, eosinophils, and neutrophils, infiltrate the wound within the first hours after injury and continue to do so for up to a week (Singer and Clark, 1999). These cells produce large quantities of reactive oxygen species and are responsible for ingestion and clearance of necrotic tissue and pathogens in the wound bed. Migration of PMNs to the wound is followed by macrophage infiltration within 1–2 days. Macrophages and Langerhans cells, dendritic cells (DCs) resident in the epidermis, are antigen presenting cells that are responsible for presenting antigens to T-cells in order to elicit an immune response. Macrophages are also responsible for producing nitric oxide (NO), an important regulator of collagen synthesis and angiogenesis, as well as many chemokines and cytokines such as Prostaglandin E_2_ (PGE_2_) and TGF-β, which induces cell proliferation and migration (Franz et al., 2007). Lastly, macrophages are essential for the initiation and propagation of new tissue formation at the wound site and facilitate the transition to the cell proliferation phase.

Thermal injury is associated with altered systemic immune function while the wound exhibits perturbed patterns of immune cell infiltration due to alterations in tissue permeability and lack of functioning vasculature in areas of coagulation (Rose and Chan, 2016). The precise alterations of immune cell infiltration in burns are not fully understood. Using murine models of flame and scald burns, Tschöp et al. demonstrated depletion of T-cells as well as decreases in the production of interferon gamma (IFN-γ) in the more severe burns, which contributed to immunosuppression by reducing the activity of the adaptive immune response. Eight days after burn, the observed immunosuppression was replaced by a predominance of a hyperinflammatory macrophage phenotype, as well as a three-fold increase in the number of IFN-γ producing T-cells. This suggests that increasing severity in burn correlates with both depressed innate and adaptive immune function (Tschöp et al., 2009). Another murine study of small TBSA (6%) burns was associated with fewer PMNs, as well as a reduced PMN respiratory burst (Calum et al., 2009).

### Immunomodulation

It is well known that immune competence is vital to proper wound healing and immune cells play a major role in combating wound infection. They also have deleterious effects if their activity in the wound microenvironment delays or prevents healing, thus yielding a chronic wound (Szpaderska and DiPietro, 2005; Franz et al., 2007; Yan et al., 2012) with continued cell proliferation and scarring (Rosique et al., 2015). As such, manipulation of the immune system (e.g., immunomodulation), both systemically and locally to enhance healing while also avoiding infection is a tempting target, it must be approached with caution.

PMNs represent an early target for cellular immune modulation as they are present in the wound immediately after injury. Application of Biafine, a topical, trolamine-containing oil-in-water emulsion, to rat burn wounds was associated with improved healing outcomes through a reduced number of neutrophils and increased macrophage numbers. The authors hypothesized that this resulted in a significant increase in the production of NO in the burn wound microenvironment, thereby increasing the rate of cell proliferation and collagen deposition (Krausz et al., 2015). Another attractive target for immunomodulation is DCs. Vinish et al. were able to control the rate of wound closure through the transient depletion or enhancement of DCs in a murine model. Depletion of DCs with Diphtheria Toxin prior to burn delayed early wound closure and formation of GT, while lowering levels of TGF-β1 and CD31^+^ blood vessels. Conversely, enhancing DC numbers with recombinant fms-like tyrosine kinase-3 ligand (Flt-3), resulted in early wound closure, increased TGF-β1, and increased vascularization in the burn wound area, without excessive deposition of collagen (Vinish et al., 2016). Based upon these findings, stimulating TGF-β1 production appears to be a target for immunomodulatory therapy, but the timing of such therapy is critical. TGF-β1 induces inflammation early in the wound healing phase, leading to a self-limiting recruitment of immune cells, followed by cell proliferation, and re-epithelialization. However, once the wound has progressed to the remodeling phase, high TGF-β1 is associated with increased scar formation (Han et al., 2012; Gilbert et al., 2016).

Non-steroidal anti-inflammatory drugs (NSAIDs) and cyclooxygenase-2 (COX-2) inhibitors have been investigated as a method to attenuate the inflammatory response (Szpaderska and DiPietro, 2005); however, it appears that route of administration may drastically affect wound healing outcomes. When administered systemically, COX-2 inhibitors were shown to reduce epithelial cell proliferation and deposition of ECM and collagen which delayed wound healing (Fairweather et al., 2015). While there have been publications on the use of COX-2 inhibitors in both animal models and clinical studies, it is difficult to say whether systemic or local NSAID therapy would be beneficial to burn wound outcomes. *In vivo* rodent models utilizing NSAID therapy focused on survival following burn infection and sepsis (Shoup et al., 1998; Schwacha et al., 2002), while most rodent studies and human clinical studies investigating NSAIDs generally focus on pain alleviation and reduction of systemic inflammation following COX-2 inhibition (Chong et al., 2014; Rose and Chan, 2016).

### Topical therapeutics for acute bacterial infection

Along with increasing rates of antibiotic resistance, the inability of systemic antibiotics to perfuse the compromised vasculature of burn wounds and penetrate the infected eschar has resulted in decades of research on novel agents and topical treatments. Comprehensive reviews of topical antimicrobial treatments for burn wounds have been recently published (Dai et al., 2010; Sevgi et al., 2013; Cartotto, 2017; Norman et al., 2017). Topical delivery of antibiotics directly to the site of injury is not a novel concept with many products already available on the market (Table 5). Topical antibiotic creams and ointments, antimicrobial impregnated dressings, and silver-based therapeutics dominate infected burn pre-clinical porcine models and clinical research (Table 6). Current research focuses on sustained delivery while maintaining bioactivity in order to reduce dressing changes, in turn reducing patient pain and burden on providers. Sustained delivery of antibiotics can be achieved by encapsulation into different hydrogel-based systems such as gelatin (Nunes et al., 2016), keratin (Roy et al., 2016), or chitosan (Hurler et al., 2012). Antibiotic incorporation into electrospun dressings (Chen et al., 2016; Dhand et al., 2016, 2017) and occlusive dressings (Steinstraesser et al., 2011) has also shown superior activity and accelerated wound healing when compared to current clinical silver-based products.

**Table 5:** FDA approved therapies for management of infection.

**Treatment type**	**Product, company**	**Indication**	**Composition**	**Format**	**FDA approval**
**INFECTION MANAGEMENT**					
	Silver sulfadiazine	For the prevention and treatment of wound sepsis in patients with 2nd and 3rd degree burns	1% micronized silver sulfadiazine	Petrolatum based cream	NDA
	Bacitracin®, Pharmacia and Upjohn	First aid to help prevent infection in minor cuts, scrapes, and burns	Mixture of related cyclic antibiotic peptides	Petrolatum based cream	NDA
	Sulfamylon®, Mylan Institutional	Adjunctive antimicrobial therapy of patients with 2nd and 3rd burns	Mafenide acetate	Water miscible cream and as a 5% solution	NDA
	Bactroban®, GlaxoSmithKline	The treatment of secondary infected traumatic skin lesions due to susceptible isolates of *S. aureus* and *S. pyogenes*	Mupirocin calcium	Cream with ~2% mupirocin calcium	NDA
	Nystatin Cream	For treatment of cutaneous mycotic infections caused by *C. albicans* and other susceptible Candida species	Nystatin	Oil based cream	ANDA
	Amphotericin B	Emperical therapy for presumed fungal infections in febrile, neutropenic patients. Treatment of patients with Aspergillus species, Candida species and/or Cryptococcus species infections	Amphotericin B	Lyophilized for injection	ANDA
	Cancidas®, Merk & Co, Inc.	Emperical therapy for presumed fungal infections in febrile, neutropenic patients. Treatment of invasive Aspergillosis in patients who are refractory to or intolerant of other therapies	Caspofungin acetate	Lyophilized and reconstituted for injection	NDA
	Vfend®, Pfizer, Inc.	For Candida infections in skin	Voriconazole	Oral and IV	NDA
**ANTIMICROBIAL DRESSINGS**					
Petrolatum coated gauze	Xeroform™ Wound Dressing, Covidien; Adaptic™ Non-Adhering Dressing, Acelity	As an initial layer in dressing wounds such as skin graft recipient sites, newly sutured wounds, and minor or partial thickness burns. It may also be used as an initial layer in dressing surgical wounds with light exudate where protection from contamination and/or deodorization is desired	Gauze or mesh impregnated with petrolatum (Xeroform includes 3% bismuth tribromophenate)	Available in strips, sheets, or rolls	510 (k)
Polyhexamethylene biguanide (PHMB)	Telfa® AMD Non-Adherent Dressing, Covidien; Tielle™ PHMB Dressing, Acelity; Kerlix™ AMD Gauze, Covidien	For use as a primary wound contact dressing or as a secondary dressing to protect against bacterial proliferation	Perforated mylar film with absorbent core, gauze, or foam dressing impregnated with PHMB	Available in strips, sheets, or rolls	510 (k)
Silver	Mepilex® Ag, Molnlycke; Silverlon®, Argentum Medical; Acticoat™, Smith & Nephew; Aquacel® Ag, ConvaTec; Allevyn™ Ag, Smith & Nephew	(1) May be used for more serious wounds such as surgical wounds or traumatic wounds left to heal by secondary intent, and partial thickness burns, wounds that are prone to bleeding, and management of painful wounds. (2) For the management of infected wounds	Variety of mesh or foam dressings with ionic silver	Available in strips, sheets, or rolls	510 (k)
Honey	MediHoney®, DermaSciences/Integra; Manukamed®, ManukaMed	Topical dressing for 1st and 2nd degree burns, skin grafts and donor sites	100% Leptospermum (Manuka) honey	Gel, paste, hydrogel sheets, impregnated gauze	510 (k)

*NDA, New Drug Application; ANDA, Abbreviated New Drug Application*.

**Table 6:** Clinical trials of therapies for management of infection.

**Clinical trial #**	**Clinical trial title**	**Intervention**	**Characteristics**			
			**Enrollment**	**Model**	**Allocation**	**Phase**
NCT02109718	A trial comparing the efficacy and safety of open dressing with petrolatum jelly vs. standard gauze dressing with silver sulfadiazine	Drug: open dressings with petrolatum jelly Drug: Silver sulfadiazine gauze dressing group	50	PA	R	3
NCT01553708	Effect of EGF with silver sulfadiazine cream compared with silver zinc sulfadiazine cream for treatment of burn wound	Drug: Epidermal growth factor with silver sulfadiazine cream Drug: Silver zinc sulfadiazine cream	34	PA		2, 3
NCT00586729	Vashe® Wound Therapy Study	Device: Vashe®Drug: Mafenide acetate	23	PA	NR	–
NCT00668044	Ciprofloxacin on burned patients	Drug: Ciprofloxacin (BAYO9867)	18	PA	NR	3
NCT02269969	Once daily aminoglycoside pharmacokinetics and optimal dosing in the burn population: a prospective study	Drug: Tobramycin	10	SGA	–	1, 2
NCT03248154	Biofilm infection in adults and children burn injury	Device: Procellera	300	PA	R	–
NCT01519492	A study of safety, tolerability, and efficacy of AFN-12520000 in the treatment of acute bacterial skin and skin structure infections due to staphylococci	Drug: AFN-12520000	103	SGA	–	2
NCT01499277	Evaluation of ceftaroline fosamil vs. vancomycin plus aztreonam in the treatment of patients with skin infections	Drug: Ceftaroline fosamil Drug: Vancomycin Drug: Aztreonam	802	PA	R	3
NCT00462904	Pharmacokinetic response to BPI in burns	Drug: Opebacan	6	SGA	NR	2
NCT00077675	Phase 2 trial of TD-6424 (Telavancin) vs. standard therapy for complicated gram positive skin and skin structure infections (gram positive cSSSI) (FAST2)	Drug: Telavancin Drug: Vancomycin or antistaphylococcal penicillin	201	PA	R	2
NCT02872272	Amikacin pharmacokinetic profile in plasma and tissue after an administration using impregnated dressings in burned patient population (AMIKACINE)	Drug: Treatment with Amikacin	75	SGA	–	4
NCT01534858	A prospective, descriptive cohort study with Prontosan+ Wound Gel X in partial and full thickness burns requiring split thickness skin grafts	Device: Prontosan® Wound Gel X	51	SGA	–	–
NCT00656708	Kerlix™ gauze study in a burn trauma unit and its effect on healthcare associated infections in burn patients	Other: Kerlix™ AMD gauze	108	SGA	–	–
NCT01439074	Mepilex+ Ag vs. silver sulfadiazine in children and adults with burn injuries	Device: Mepilex® Ag Drug: Silver Sulphadiazine Ag cream	162	PA	R	–
NCT00742183	Evaluating the cost-effectiveness, efficacy, safety and tolerance of Mepilex® Ag vs. Silvadene®	Device: Mepilex® Ag Device: Silvadene	100	PA	R	–
NCT02210208	A soft silicone wound contact layer containing silver in the treatment of skin grafts in surgical burn patients. (MpTAg03)	Device: Mepitel® Ag Device: Mepilex® Transfer Ag	25	PA	NR	–
NCT01214811	Open multi-center investigation to evaluate signs and symptoms of local inflammation/infection on chronic ulcers and partial thickness burns when using mepilex border ag as an anti-microbial Wound Dressing	Device: Mepilex® Border Ag	27	SGA	–	3
NCT02681757	Comparison of Mepitel Ag vs. antibiotic ointment used with soft cast technique for treatment of pediatric burns	Device: Triple antibiotic ointment dressing Device: Mepitel® Ag	100	PA	R	4
NCT02852148	ACTICOAT™ for the treatment of burns and chronic wounds	Device: ACTICOAT™	25	SGA	–	–
NCT01598493	To study the healing effect of silver impregnated activated carbon fiber wound dressing on deep dermal burn	Device: biomedical carbon technology antimicrobial dressing Drug: Flamazine	30	PA	R	–
NCT03048188	Manuka honey in second- and grafted third-degree burns	Other: Wound dressing	10	SGA	–	–

–*, study did not mention; Ag, Silver; SOC, Standard of Care. Model: PA, Parallel Assignment (Therapy vs. SOC); SGA, Single Group Assignment. Allocation: R, Randomized; NR, Non-Randomized*.

Natural antimicrobial products are once again taking the forefront of antimicrobial therapies (Newman and Cragg, 2012; Bitter and Erickson, 2016) due to their wide availability and inexpensiveness compared to current SOC. For instance, medicinal honey-based therapeutics are currently being investigated due to their antimicrobial and wound healing properties. In addition, overall patient satisfaction is reported to be higher when using medicinal honey compared to SSD (Nasir et al., 2010; Aziz and Abdul Rasool Hassan, 2017). There are a number of different varieties of honey based on the plant-derived active ingredients, but the most well-known is sourced from the Manuka tree in New Zealand (Carter et al., 2016; Duncan et al., 2016). In a RCT of 150 patients, two similar burn injuries were chosen on different parts of the patient's body and randomized to treatment with honey and the other with SSD. Honey accelerated re-epithelialization and had a lower infection rate compared to SSD (Malik et al., 2010). Another potential natural therapy uses medicinal herbs such as *Centella asiatica* incorporated into topical ointments (Centiderm®). The active triterpene glycosides within Centiderm® transforms by hydrolysis into asiatic acid, which has shown to reduce the incidence of wound infections. In a recent clinical study, no infections were observed in the Centiderm®-treated group of 40 burn patients while 4 of 35 patients in the SSD group developed infections at the treatment site (Saeidinia et al., 2017). In addition, topical oak bark ointment successfully reduced the quantity of Methicillin-resistant *Staphylococcus aureus* (MRSA) pathogens in a mature infection when applied twice daily when compared to SSD (Davis and Mertz, 2008).

The antimicrobial properties of silver-based dressings have been utilized for a number of FDA approved burn dressings. Unfortunately, a number of adverse outcomes are observed when using silver-based dressings such as delayed or incomplete re-epithelialization, scar discoloration, and hypersensitivity (Hussain and Ferguson, 2006; Wang et al., 2009a). For this reason, novel delivery systems for silver-based therapeutics have been developed including silver-loaded hydrogels (Boonkaew et al., 2014), which have found success in clinical applications (Glat et al., 2009). Even though there was no difference in infection rate between the silver-loaded hydrogel and SSD, there was a decrease in patient pain during dressing changes (Glat et al., 2009). In one report, a hydrofiber dressing coated with ionic silver reduced the incidence of burn wound infections, reduced pain, and accelerated wound closure when compared to SSD. The hydrofiber dressing was changed every 3 days unlike SSD cream which requires daily dressing changes (Muangman et al., 2010). Due to the limitations of silver-based products, a number of alternative metals with antimicrobial properties, such as copper and gallium, are currently being investigated as well (Sevgi et al., 2013).

Antimicrobial peptides (AMPs) are commonly cationic and have broad-spectrum antimicrobial activity, targeting bacterial cell membranes and disintegrating their lipid bilayer structure (Mahlapuu et al., 2016). Even though AMPs are currently not in clinical trials for burns, they have therapeutic potential in a range of infections, including those producing biofilms (Findlay et al., 2016; Ma et al., 2017). Topical application of epinecidin-1 to MRSA contaminated porcine burn wounds prevented infection, sepsis, and delayed wound healing (Huang et al., 2017). The treatment was administered 6 h after wound inoculation; therefore, further testing within a mature infection would highlight the potential of this modality as a therapy in addition to being a preventative measure.

Instead of focusing on antimicrobials to combat infectious microorganisms, current research also investigates the ability of predatory and probiotic bacteria to suppress colonization of pathogenic bacteria such as *Pseudomonas* (Kadouri et al., 2013). Recently, a clinical trial of 80 burned patients were treated with the probiotic bacteriotherapy through topical application of the *Lactobacillus* genus. When compared to SSD treatment, the probiotic decreased overall infection rates and promoted GT deposition (Peral et al., 2009). As we continue our understanding of the burn wound microbiome and the events to why some contaminations develop to be invasive infections, novel bacteriotherapies will arise.

The biofilm of a wound, much like an eschar, often prevents antimicrobial agents from reaching the wound bed (Phillips et al., 2015). For this reason, biofilm disrupting agents (synthetic and natural) are an emerging class of therapeutics used to penetrate and destabilize the biofilm microenvironment, leaving pathogens vulnerable to antimicrobial activity. Aryl-alkyl-lysines are small molecules that have been successful against both planktonic as well as the mature biofilm of an *Acinetobacter baumannii* burn wound infection (Ghosh et al., 2016). Also, a formulated garlic ointment has been capable of preventing biofilm development as well as disruption of immature biofilms with a spectrum of activity against many common burn pathogens (Nidadavolu et al., 2012).

### Topical therapeutics for acute fungal infection

The increased prevalence of fungal wound infections has spurred research and development of novel antifungal treatments. *Candida albicans* is the most common fungus to infect burn wounds and represents the major target of current pre-clinical research. Silver-coated dressings and Nystatin have proven to be effective treatments (Acar et al., 2011). Electrospun SSD-containing nanofiber dressings were also shown to be effective against *C. albicans* infected burn eschar (Ciloglu et al., 2014). In many burn centers, systemic administration of Amphotericin B is used when invasive fungal infection is suspected; however, systemic administration of Amphotericin B is associated with a dose-dependent nephrotoxic effect (Hamill, 2013). This has led to the development of topically applied Amphotericin B encapsulated in polyethylene glycol and chitosan. Sustained release of Amphotericin B from the nanoparticle was able to clear fungal infections while having no adverse effect on wound healing (Sanchez et al., 2014). Even though topical application of Amphotericin B reduces the overall dose, Amphotericin B release from a carrier to the blood stream could potentially result in systemic dispersion and toxicity. For this reason, topical therapeutics using Amphotericin B must be evaluated for any signs of nephrotoxic effects.

The current approved therapies from the management of infection (Table 5) consists of either drugs that required a clinical trial or 510 (k) approved dressings that contain a well know antimicrobial (PHMB or silver). In current clinical trials are a wide range of new TERM strategies that are being explored that will require similar approval mechanisms (Table 6). What is still missing is comprehensive RCT of these agents comparing efficacy to each other (i.e., the silver dressings).

## Cell proliferation

Wound closure, generally accepted as complete re-epithelialization, is the purpose of the cell proliferation phase. Re-establishing the epidermal layer is paramount in regenerating the protective barrier of skin, preventing infection, and limiting fluid loss. Within days of injury, fibroblasts migrate into the wound and deposit large amounts of ECM consisting first of relatively disorganized type III collagen with wound collagen content reaching its peak 2–3 weeks after injury. These fibroblasts often differentiate into myofibroblasts which possess a contractile phenotype and are easy to identify due to their expression of α-SMA (alpha smooth muscle actin). New blood vessels are formed by invading endothelial cells throughout the ECM to supply nutrients to the newly forming GT. As the wound fills in with GT, keratinocytes at the wound edges migrate and proliferate over the top of the wound until wound closure takes place (Werner and Grose, 2003).

In uninjured skin, the basal epithelial cells self-renew and constantly differentiate into the epidermis every 2–3 weeks. The epidermis also contains endogenous stem cells that respond immediately to an injury and start the self-repair process. Without the basal epithelial cells in the epidermis, the hair follicle stem cells (HFSCs) that reside at the base of the hair follicle act as foci for re-epithelialization. Hair follicles extend through the entire depth of the dermis and some viable HFSCs are present even in DPT wounds. In the event of a PT or deeper burn, the entire epidermis is lacking and thus an epidermal replacement is needed. For FT burns, the entire dermis has also been lost and needs to be replaced; otherwise, the resulting quality of life may be impacted by contractures and scarring (Singer and Clark, 1999; Werner and Grose, 2003; Diegelmann and Evans, 2004).

### Burn wound coverings

According to a panel of experts from American Burn Association, the burn wound coverings are classified under two broad categories, *Skin replacement*: defined as a tissue or graft that permanently replaces lost skin with healthy skin, and *Skin Substitute*: defined as a biomaterial, engineered tissue, or combination of materials and cells or tissues that can be substituted for skin autograft or allograft in a clinical procedure (Kagan et al., 2013). Currently available burn wound products fall under either of the above mentioned class of wound dressing categories. The burn wound coverings can be further divided into temporary biological coverings, epidermal, dermal or complete skin substitutes (Tables 7, 8).

**Table 7:** HCT/P products approved for burns.

**Banked Human tissue: allograft (amniotic membrane)**		**Allograft intended for use as a biological membrane covering for PT and FT acute wounds such as burn**
**Treatment type**	**Product, company**	**Composition**
Cellular	Affinity™, Organogenesis, Inc.	Fresh AM with viable cells stored hypothermically
	Grafix®, Osiris Therapeutics, Inc.	Cryopreserved sheets stored at −75 to −85°C for 2 years
Acellular	ActaShield™ Amniotic Barrier Membrane, Wright Medical Technology	Decellularized, dehydrated sheets stored RT for 5 years
	AmnioBand®, Musculoskeletal Transplant Foundation	Dehydrated sheets stored RT for 3 years
	AmnioFix® and EpiFix®, MiMedx Group	Dehydrated sheets stored RT for 5 years
	Amnioshield®, Alphatec Spine, Inc.	Dehydrated sheets stored RT for 5 years
	Biovance®, Alliqua Biomedical	Decellularized, dehydrated sheets stored RT
	Dermavest™, Aedicell	Decellularized AM ECM Particulate pressed into a pad that can be stored at RT for 3 years
	Clarix™, and Neox®, Amniox® Medical	Cryopreserved umbilical cord and AM sheets available cryopreserved and fully hydrated at RT for 2 years
	NuShield™, Organogenesis, Inc.	Sterilized, dehydrated sheets stored at RT
	Revitalon™, Medline Industries	Aseptically processed sheets stored at RT
**Banked human tissue: allograft (human skin)**		**Allograft intended for use as a biological membrane covering for PT and FT acute wounds such as burn; or for repair or replacement of damaged or inadequate integument tissue or for other homologous uses of human integument**
Cellular	Burn Care Allografts, Allosource	Bilayered allograft consisting of viable cells in epidermis and dermis available in fresh and cryopreserved solid and meshed sheets
	ReadiGraft®, LifeNet Health	Same as above; only available in cryopreserved sheets
	Theraskin®, SolSys™ Medical	Same as above; only available in cryopreserved sheets
Acellular	AlloDerm® Regenerative Tissue Matrix, LifeCell Corp.	Acellular, FD dermal sheets stored at RT
	AlloPatch®, Musculoskeletal Transplant Foundation	Acellular dermal hydrated sheets stored at RT for 3 years
	DermaCell®, LifeNet Health	Decellularized dermal solid and meshed hydrated sheets stored at RT
	DermaPure™, Tissue Regenix Wound Care	Decellularized dermal sheets stored at RT
	DermaMatrix™, Synthes	FD non-cross-linked dermal sheets stored at RT
	FlexHD®, Ethicon	Acellular hydrated non-sterilized dermal sheets at RT
	GammaGraft™, Promethean LifeSciences, Inc.	γ-Irradiated skin
	GraftJacket™ Regenerative Tissue Matrix, Wright Medical Technology	Acellular, FD dermal sheets
	Maxxeus™ Skin, Community Tissue Services	Cryopreserved low dose terminally sterilized skin

*FD, Freeze Dried; FT, Full-Thickness; PT, Partial-Thickness; RT, Room Temperature*.

**Table 8:** FDA cleared and approved epidermal, dermal and complete substitutes for burn wounds.

**Treatment type**	**Product, company**	**Indication**	**Composition**	**Format**	**FDA approval**
**EPIDERMAL SUBSTITUTES**					
Cellular	Epicel®, Vericel Corporation	DPT or FT burns over a TBSA ≥30%. It may be used with STSG, or alone in patients for whom STSG may not be an option due to the severity and extent of their burns	Autologous epithelial cells grown with mouse fibroblasts to form a CEA	Grafts are attached to petrolatum gauze and delivered in temp controlled packaging that are viable for 24 h	HDE
**DERMAL SUBSTITUTES: XENOGRAFT**					
Acellular	EZ Derm®, Molnlycke Health Care	Use for burns and donor sites reduces pain and fluid loss. Can also be used as a temporary cover, or test graft, prior to autografting and as a protective covering over meshed autografts	Acellular porcine dermis	Solid and meshed in rolls, patches, or sheets stored at RT	510 (k)
	PriMatrix™, Integra LifeSciences	For management of 2nd degree burns and donor sites/grafts	Acellular fetal bovine dermis	Solid, fenestrated, and meshed sheets stored at RT for 5 years	510 (k)
	MariGen™ Wound Dressing, Kerecis Limited	Same as above	Acellular cod fish dermis	Solid and meshed sheets stored at RT	510 (k)
	Oasis™ Wound Matrix, Cook Biotech	Same as above	Acellular freeze-dried porcine small intestinal mucosa	Available in a single layer or tri-layer sheet stored at RT for 2 years	510 (k)
	Helicoll™, Encol Corp.	Same as above	Bovine dermal derived acellular collagen matrix	Hydrated sheets stored at RT for 3 years	510 (k)
	MatriStem® and Cytal® Wound Matrix, Acell, Inc.	Same as above	Porcine derived ECM scaffolds from urinary bladder matrix	Dehydrated single and multi-layer sheets with or without fenestrations, also in flowable	510 (k)
	Endoform™, Hollister Wound Care	Same as above	Naturally derived ovine collagen ECM	Solid or fenestrated sheets stored at RT	510 (k)
	PuraPly™ Antimicrobial Wound Matrix, Organogenesis, Inc.	Same as above	Cross-linked porcine intestinal collagen coated with 0.1% PHMB	Sheets stored at RT	510 (k)
	Suprathel® wound and burn dressing, polymedics innovations	Same as above	Synthetic copolymer of polyactide, trimethylene carbonate, and s-caprolactone	Sheets stored at RT	510 (k)
	Hyalomatrix®, Medline Industries, Inc.	Same as above	Esterified hyaluronic acid scaffold on a semipermeable silicone outer layer	Sheets stored at RT	510 (k)
	Architect®, Harbor MedTech	Same as above	Stabilized ECM collagen matrix (>95% Type I) derived from equine pericardial	Solid and fenestrated decellularized, dehydrated sheets stored at RT	510 (k)
	Integra® Dermal Regeneration Template, Integra Life Sciences	Post-excisional treatment of life-threatening DPT and FT thermal injuries where sufficient autograft is not available at the time of excision or not desirable	Chemically cross-linked bovine collagen and GAGs with and without on a semipermeable silicone backing	Hydrated solid and meshed sheets stored at RT; Also available in a flowable format	PMA
	Biobrane®, Smith and Nephew	Temporary coverage of PT burns once debrided, excised burn wounds with or without meshed autografts, and donor sites	Biosynthetic wound dressing of a fine nylon mesh cross-linked with porcine dermal collagen on a semipermeable silicone backing	Dry sheets or glove design stored at RT	510 (k)
Cellular	TransCyte™ (previously called Dermagraft-TC) Organogenesis, Inc.	For use as a temporary wound covering for surgically excised FT and DPT thermal burn wounds in patients who require such a covering prior to autograft placement; and for the treatment of mid-dermal to indeterminate depth burn wounds that typically require debridement and that may be expected to heal without autografting	Allogeneic neonatal human foreskin fibroblasts grown on nylon mesh combined with a silicone layer	Cryopreserved cells on nylon mesh	PMA
**COMPLETE SUBSTITUTES: (EPIDERMAL-DERMAL COMBINATION)**					
Cellular	OrCel™, Forticell Bioscience, Inc.	For the treatment of fresh, clean split thickness donor site wounds in burn patients	Bilayer cellular matrix in which normal human allogeneic neonatal keratinocytes and dermal fibroblasts are cultured in two separate layers into a Type I bovine collagen sponge	Sheets that are stable for 72 h in a temperature-maintained shipping container	PMA

*CEA, Cultured Epithelial Autograft; DPT, Deep Partial-Thickness; ECM, Extracellular Matrix; GAG, Glycosaminoglycan; FT, Full-Thickness; HDE, Humanitarian Device Exemption; PHMB, Polyhexamethylene biguanide; PMA, Pre-market Approval; RT, Room Temperature; STSG, Split-Thickness Skin Graft*.

#### Temporary biological coverings

Allografts are used as temporary biological coverings which serve as lifesaving treatments for patients with extensive burns and limited donor skin (Brown et al., 1953; Zuo et al., 2017). These coverings are utilized to provide barrier function to prevent bacterial infection and provide thermoregulation (Mohammadi et al., 2013). With advancements in preservation processes, human allografts (derived from amniotic membrane (AM) or skin) can be stored and banked sterilely, either with viable cells or as a decellularized product with a plethora of human -based products commercially available for burn wound coverage (Table 7). Human AM is considered an effective biological material due to its unique composition of substrate proteins, specifically collagen IV, laminin, integrin, and proteoglycans, and it has been proposed to benefit burn wound healing specifically (Kesting et al., 2008; Glat and Davenport, 2017; Mowry et al., 2017; Tenenhaus, 2017). Fresh AM demonstrated higher graft take compared to autograft on burn wounds (Mohammadi et al., 2013) while glycerol preserved or air-dried AM reduced the time to re-epithelialize on autograft donor sites (Zidan et al., 2015) and burn wounds (Singh and Chacharkar, 2011), respectively. A recent review shows a series of case studies using AM allograft, to treat PT and FT burns in various anatomical locations to promote wound re-epithelialization and vascular angiogenesis (Reilly et al., 2017). Current clinical trials further characterize donated amnion as a skin substitute for burn patients (Table 9); though the results are not posted, many anticipate AM will prove to be a safe and efficacious temporary biological covering.

**Table 9:** Clinical trial therapies testing skin substitutes for burn wounds.

**Clinical trial #**	**Clinical trial title**	**Intervention**	**Characteristics**			
			**Enrollment**	**Model**	**Allocation**	**Phase**
NCT02904941	Human amniotic vs. synthetic membrane as a transient skin cover for pediatric burns	Biological: AM Dressing Device: Synthetic Dressing Procedure: SOC	60	PA	R	–
NCT02765737	Dehydrated human amnion chorion membrane (dHACM) vs. control in the treatment of partial thickness burns	Other: Dehydrated AM Device: Mepilex® Ag	60	PA	R	–
NCT01454310	An acellular epithelial skin substitute in deep partial-thickness burns	Device: Wound coverage by acellular skin substitute Device: STSG	18	PA	NR	4
NCT02994654	CONTINUED ACCESS PROTOCOL: demonstration of the safety and effectiveness of ReCell® combined with meshed skin graft for reduction of donor area in the treatment of acute burn injuries	Device: ReCell®	60	PA	R	–
NCT03333941	Continued access to the Recell® device for treatment of acute burn injuries	Device: ReCell® autologous cell harvesting device	60	Ob	–	–
NCT02380612	ReCell® combined with meshed skin graft in the treatment of acute burn injuries	Device: ReCell® Treatment Procedure: meshed STSG	30	PA	R	–
NCT02992249	Prospective evaluation of the ReCell® autologous cell harvesting device for specific compassionate use cases	Device: ReCell®	68	Case	–	–
NCT02905435	Assessment of safety and effectiveness of biodegradable temporizing matrix in the treatment of deep burn skin injuries	Device: Biodegradable Temporizing Matrix	10	SGA	–	–
NCT00548314	Dermal substitute and topical negative pressure in burns (VAC-M)	Other: Dermal matrix (Matriderm®) Procedure: STSG Device: VAC® therapy (KCI)	86	PA	R	3
NCT03077087	Single-stage integra reconstruction in burns (Integra®)	Device: Thin Integra®	10	SGA	–	–
NCT02982096	Study comparing healing with epidermal fractional blister grafting (Cellutome™) to a cellular technique	Device: Cellutome™ Device Other: SOC	30	PA	R	–
NCT01512017	Clinical study on Veloderm® for the treatment of split-thickness skin graft donor sites	Device: Crystalline cellulose simple occlusive dressing Device: Vaseline	96	PA	R	3
NCT02350205	SASS 2 : Self assembled skin substitute for the autologous treatment of severe burn wounds in acute stage of burn trauma	Biological: Self assembled skin substitute (SASS) Procedure: STSG	17	CA	R	1, 2
NCT03227146	Study with an autologous dermo-epidermal skin substitute for the treatment of burns in adults	Biological: EHSG-KF Biological: STSG	12	SGA	–	2, 3
NCT03229564	Study with an autologous dermo-epidermal skin substitute for the treatment of burns in children	Biological: EHSG-KF Biological: STSG	12	SGA	–	2, 3
NCT01655407	Safety and efficacy study of autologous engineered skin substitute to treat partial- and full-thickness burn wounds	Drug: Autologous Engineered Skin Substitute Drug: STSG	10	PA	R	2
NCT00718978	Clinical application of autologous three-cellular cultured skin substitutes (CSS)	Procedure: CSS grafting	11	SGA	–	–
NCT02145130	Phase I study for autologous dermal substitutes and dermo-epidermal skin substitutes for treatment of skin defects	Biological: denovoDerm Biological: denovoSkin	20	PA	NR	1
NCT00618839	StrataGraft® skin tissue (human donor skin) in the surgical management of complex skin defects	Biological: StrataGraft® Skin Tissue Procedure: Cadaver allograft	15	SGA	–	1, 2
NCT01437852	StrataGraft® skin tissue as an alternative to autografting deep partial-thickness burns	Biological: StrataGraft® Skin Tissue	30	SGA	R	1

–*, study did not mention; SOC, Standard of Care; STSG, Split-Thickness Skin Graft. Model: CA, Crossover Assignment; Ob, Observational; PA, Parallel Assignment (Therapy vs. SOC); SGA, Single Group Assignment. Allocation: R, Randomized; NR,– Non-Randomized*.

#### Epidermal substitutes

Epithelial cells applied either as sheets or as a spray represent the most common epidermal substitutes. Autologous epithelial cells obtained from a small biopsy of a patients' own skin have been successfully grown on a mouse irradiated fibroblast feeder layer and used to treat large TBSA burn injury. These sheets of cells are known as cultured epithelial autografts (CEA). The applicability of CEA to burn wounds was widely recognized following the introduction of Epicel™ in 1988. Since then, many studies show the benefits of CEA in providing coverage to extensive burn wounds (Wood et al., 2006; Sood et al., 2010; Cirodde et al., 2011). CEA success in the literature is variable, largely due to their delicate nature (only 7–10 cell layers of keratinocytes), need for an uninfected wound bed, expansion time for cells, and issues with transfer of the graft. To address these limitations, production of CEA on a chemically defined surface for easy and quick transfer to the wound bed has been developed (Myers et al., 1997; Wright et al., 1998; Horch et al., 2000; Hernon et al., 2006). Clinical usage of CEA with (1:6) widely meshed autografts demonstrated similar healing outcomes to (1:3) meshed autografts (Akita et al., 2018).

Similarly, suspensions of autologous cells (including epidermal progenitors and basal epithelial cells) applied with a spray device produce acceptable clinical outcomes (Yim et al., 2011; Esteban-Vives et al., 2016). A newer technology, “ReCell®,” which is an epithelial spray preparation device, has gained popularity applying autologous cells to the wound bed (Table 9). Using this device, epithelial cells can be isolated from a small biopsy of the patients' own skin and sprayed directly on the burn wound after excision or applied along with meshed autograft (Gravante et al., 2007; McHeik et al., 2014).

#### Dermal substitutes

CEA and suspended epidermal cell technologies lack a vital component of skin—the dermis. Artificial dermal products were designed to significantly reduce the time needed to achieve final wound closure in the treatment of major burn wounds. This process typically requires a two-stage method with the first to apply the skin substitute in order to create a wound bed of GT, and the second to apply an autologous graft on the neodermis. One such clinical study used Integra® after early excision of burned tissue and was allowed to integrate. After 3 weeks, CEA was applied on top of the resulting wound bed. The reconstructed skin was durable with no signs of dehiscence (Matsumura et al., 2013). Recently, treatment of complex FT soft tissue injuries with Integra® combined with ReCell® reduced donor site skin requirements, permitted wider meshed autografts, and reduced time to complete healing (Hammer et al., 2017). Use of Integra® seeded with adipose tissue derived stem cells (ASCs) in porcine FT burns enhanced wound angiogenesis, blood vessel maturation, and matrix remodeling compared to Integra® without cells (Foubert et al., 2015). Currently, Integra® is being investigated as an adjunct to a meshed autograft in a single-stage surgery (Table 9). Newer techniques to incorporate stem cells or macromolecules, such as tropoelastin (Wang et al., 2015), may improve the efficiency of Integra®.

Parallel to Integra®'s development, Biobrane® was developed as a bilaminate membrane with an ultrathin layer of silicone rubber mechanically bonded to a knitted nylon fabric outer layer and porcine type I collagen inner layer into which GT grows (Frank et al., 1984; Yang et al., 1989). A recent retrospective study showed that application of Biobrane® maintains a healthy wound bed after burn excision and prior to grafting (Tan et al., 2015). Furthermore, Biobrane®, has been shown to decrease pain and hospitalization in PT burns (Lal et al., 2000). A recent study comparing Biobrane® to allograft for temporary coverage determined Biobrane® to have a lower cost and significantly reduced procedure time (Austin et al., 2015).

Many other synthetic dermal substitutes were introduced following the success of Integra® and Biobrane® (Table 8). In order to closely mimic the structural architecture and retain the biomolecular composition of dermis, specifically collagen, attempts were made to use completely decellularized skin tissue. The process of decellularization isolates the ECM scaffold of a tissue by chemically removing cells, yielding less immunogenic substrates for tissue regeneration (Chen et al., 2004; Gilbert et al., 2006). Many xenograft dermal substitutes are produced via such a process from animal skin (porcine, ovine, or bovine) and are indicated to treat PT burn injuries (Table 8). Apart from biological and biosynthetic dermal matrices, completely synthetic dressings like Suprathel®, were developed to cover burn wounds and has demonstrated equal efficacy as Biobrane® when applied over PT burns (Rahmanian-Schwarz et al., 2011). The concept of using dermal substrates to grow and deliver fibroblast was long realized when TransCyte™ (formerly marketed as Dermagraft-Transitional Covering) was introduced (Noordenbos et al., 1999). A prospective RCT using Dermagraft-TC™/TransCyte™ in patients with PT burns demonstrated faster re-epithelialization and fewer dressing changes compared to patients treated with Biobrane® or Silvadene® (Kumar et al., 2004).

The next generation skin graft may currently be in development in the form of genetically modified porcine skin [α-1, 3-galactosyltransferase knockout (GalT-KO)] which could significantly ease the availability of clinically acceptable xenografts (Leto Barone et al., 2015). The GalT-KO xenografts are tolerated similarly to the fresh or cryopreserved allografts (Leonard et al., 2017). If proven safe to be applied clinically, the cost may be considerably reduced and immediate availability of off-the-shelf xenograft for burn victims can be expected.

#### Complete substitutes

To date, very few epidermal-dermal “complete” substitutes have been investigated. One example is Apligraf®, which is bovine type I collagen populated with neonatal fibroblasts and seeded by living human keratinocytes. In a multicenter RCT of 40 burn patients, Apligraf® placed over meshed autograft improved cosmetic and functional outcomes (Waymack et al., 2000). Another bilayer device, OrCel™, was introduced soon after Apligraf® to treat donor sites, where it accelerated healing and reduced scarring (Still et al., 2003). It is noteworthy, that despite allogeneic and synthetic origin of Apligraf®, OrCel™, and Dermagraft-TC™, rejection has not been an issue.

StrataGraft®, currently in clinical trials, is produced using NIKS® cells (human keratinocyte progenitor cell line), and is a viable FT product developed for treatment of severe burns after excision. StrataGraft® skin tissue consists of a stratified epidermal layer with a fibroblast laden collagen dermal component, is less fragile than CEA, and can be sutured, stapled or secured with an adhesive and remains intact on the wound bed, providing the critical barrier function during wound healing (Centanni et al., 2011; Table 9).

Cultured skin substitutes (CSS) contain collagen-glycosaminoglycan substrates with autologous fibroblasts and keratinocytes, and they are currently under clinical investigation as autologous engineered skin substitutes (ESS) (Table 9). CSS are proposed to provide permanent replacement of both dermal and epidermal layers in a single grafting procedure with similar mechanical properties as skin (Boyce et al., 2006; Sander et al., 2014). A recent RCT using CSS indicated that autologous ESS reduces mortality and requirements for donor skin to cover FT burns of greater than 50% TBSA (Boyce et al., 2017). Like CEA, though, CSS requires long culture times before application. In addition, CSS does not meet HCT/P designation because the cells are more than minimally manipulated, so no FDA approved indications exist presently (Table 1).

Use of dextran and fibrin hydrogels are currently being pre-clinically investigated as options to treat PT or FT burn wounds (Shen et al., 2015; Burmeister et al., 2018a). Bio-printing technology can develop three dimensional skin substitutes customized to individual patients (Ng et al., 2016). Still, the bio-printing process of skin requires autologous cells, a limitation in large TBSA burns. Despite successful skin substitute use in some arenas, further research into novel products is ongoing.

### Stem cells

Among the variety of available stem cells (Table 2), mesenchymal stem cells (MSCs) have multipotent potential and are easy to isolate, leading to their widespread adoption in wound healing literature. Recent reviews specifically address the key role of MSCs in burn wounds (Cheng et al., 2017; Maranda et al., 2017). Issues were identified that complicate comparing the efficacy of MSCs include the potential immune response, isolation procedures, culturing conditions, validation of differentiation potential, required therapeutic dose of cells, methods to deliver the cells, and the long-term viability of the cells.

Regardless of wound type, MSCs secrete anti-inflammatory factors such as IL-10 and tumor necrosis factor inducible gene siRNA6 (TSG-6) (Ennis et al., 2013). Recent studies show MSCs from different sources [bone marrow (BMSCs), ASCs, umbilical cord, and Wharton jelly] all reduced macrophage secreted pro-inflammatory cytokines IL-1α, IL-6, and IL-8 via PGE_2_ which potentially in burn wounds resolve the inflammatory phase (Najar et al., 2010; Yañez et al., 2010; Jin et al., 2013). In addition, it is worth remembering that elevated levels of NF-κB following tissue injury stimulates the secretion of PGE_2_ by MSCs which in turn significantly reduces the inflammatory cytokine surge following a burn injury.

BMSCs respond to the host chemokines: CXCR12/CXCR4, angiopoietin 1 (Ang-1), tyrosine kinase receptor, PDGF-β, and Tie-2 to facilitate MSCs-host endothelial cell interactions and wound vascularization (Lozito and Tuan, 2011; Hu et al., 2013). Further, BMSCs increase the stability of newly formed blood vessels by inhibiting high levels of exogenous matrix metalloproteinase 2 and 9 via tissue inhibitors of MMP-1 and−2 secretion (Kachgal and Putnam, 2011). Furthermore, BMSCs injected near the site of a burn wound differentiate into multiple skin cell types including keratinocytes, endothelial cells, pericytes, and monocytes. The BMSCs were traceable up to 3 months post-injury but not in 120 day mature scars, suggesting BMSCs play a role in wound healing and remodeling, but contribute less to long-term homeostasis, specifically scarring (Rea et al., 2009).

There is interest in purposing surgically discarded adipose tissue as immediate bed-side treatments. DPT and FT burn wounds treated with processed tissue improved GT formation, increased vascular endothelial growth factor (VEGF) levels at the wound site, and improved tissue re-vascularization (Atalay et al., 2014). Similarly, culture expanded ASCs implanted in the wound site responded to stromal derived factor one (SDF-1) and home to the perivascular space of nearby host blood vessels by binding to CXCR4 and CXCR7 (Stuermer et al., 2015; Kosaraju et al., 2016). To this end, a growing body of research has led to the discovery of MSCs from several different anatomical locations with each warranting further investigation into their wound healing potentials.

An alternative approach to delivering stem cells to the wounds involves collecting the products secreted from them and then applying this “secretome.” This secretome consists of extracellular vesicles, growth factors, and other proteins. The secretome profile of MSCs reveals that they express an array of pro-regenerative factors (Kilroy et al., 2007; Prockop and Oh, 2012; Phinney and Pittenger, 2017). Extracellular vesicles including exosomes and macrovesicles contain mRNA, microRNA (miRNA), and proteins which can be transferred between cells to regulate cell-to-cell communication, signaling, and altering cell or tissue metabolism. These molecules influence the response to injury and infection thereby highlighting their potential as therapeutics after burn injury (Levin and Sukhareva, 2016; O'Dea et al., 2016). In particular, MSC-derived exosomes reduced burn induced inflammation (Li et al., 2016). In a separate study, the paracrine factors from irradiated blood cells were collected. The secretome was then loaded into a commercially-available hydrogel and applied to a burn which was then covered with an autograft. Interestingly, the secretome from the irradiated cells led to improved angiogenesis in the wound when compared to the secretome from healthy cells (Hacker et al., 2016).

MSCs reduce HTS formation via constitutive paracrine effects of anti-inflammatory (PGE_2_) and anti-fibrotic factors, including hepatocyte growth factor, basic fibroblast growth factor (bFGF), and VEGF (Fang et al., 2016). Synergistically, these cytokines down-regulate expression of TGF-β1 and collagen (I and III) by fibroblasts (Zhang et al., 2006). MSCs have shown to improve long term scar outcomes when utilized early after burn injury; however, the use of stem cells after scar formation, specifically after a burn injury, still has to be explored.

A small number of trials have been conducted in the United States that utilize stem cells in the acute phase after burn with the main goal of establishing the safety of this treatment modality of which a phase 1, interventional clinical study is currently under investigation to determine if MSC treatment will improve healing and scarring of PT burns (Table 10). Thus far, most of the studies investigating the use of MSCs either applied them topically onto the wound or by injection at the wound site. New devices (e.g., hydrogels, nano-/micro-particles, nanofibers, ECMs, spheroids, and synthetic scaffolds) can deliver MSCs to maximize their potential at accelerating wound healing (Steffens et al., 2014; Chung et al., 2016).

**Table 10:** Clinical trials for stem cells.

**Clinical trial #**	**Clinical trial title**	**Intervention**	**Characteristics**			
			**Enrollment**	**Model**	**Allocation**	**Phase**
NCT02104713	Stem cell therapy to improve burn wound healing	Biological: Allogeneic (MSC's) Application to the Burn Wounds	20	SGA	–	1
NCT03113747	Allogeneic ADSCs and platelet-poor plasma fibrin hydrogel to treat the patients with burn wounds (ADSCs-BWs) (ADSCs-BWs)	Biological: ALLO-ASCs	20	PA	R	1, 2
NCT02394873	A study to evaluate the safety of ALLO-ASC-DFU in the subjects with deep second-degree burn wound	Biological: ALLO-ASC-DFU	5	SGA	–	1
NCT02619851	A clinical trial to evaluate the safety and efficacy of ALLO-ASC-DFU for second deep degree burn injury subjects	Biological: ALLO-ASC-DFU Device: Conventional Therapy	22	PA	R	2
NCT03183622	A follow-up study to evaluate the safety of ALLO-ASC-DFU in ALLO-ASC-BI-101 clinical trial	Biological: ALLO-ASC-DFU	5	Ob	Case	1
NCT02779205	Child's adipose cells: capacity of tissue regeneration (cicASChild)	Procedure: Adipose tissue sample	38	PA	NR	–

–*, study did not mention; ASC, Adipose-derived Stem Cells; MSC, Mesenchymal Stem Cells; STSG, Split-Thickness Skin Graft. Model: Ob, Observational; PA, Parallel Assignment (Therapy vs. SOC); SGA, Single Group Assignment. Allocation: R, Randomized; NR, Non-Randomized*.

According to the FDA, the use of homologous stem cells for skin regeneration—where donor cells or tissues match recipient cells or tissues and perform the same basic function(s)—is regulated separately from non-homologous cells or tissues. These non-homologous treatments are not regulated by the HCT/P exemption and instead are considered a “biologic,” requiring an IND, clinical trials showing both safety and efficacy, and BLA approval (Table 1) (FDA, 2017). With the implementation of the World Health Organization (WHO) mandate “WHO Guiding Principles on Transplantation,” it is expected that a global consensus on standard manufacturing protocols will be achieved for stem cells and future clinical trials will be performed with well-characterized cells under standardized conditions (http://www.who.int/transplantation/en/).

### Growth factors and gene therapy

To improve wound healing in burns, studies have investigated the application of growth factors derived from allogeneic sources, recombinant yeast, or bacteria. Treatment with these factors may occur alongside SOC (e.g., a skin graft). The existing body of literature on growth factor application to treat burn wounds is variable, though, with different burn pathologies and pre-clinical models studied. In contrast, a great deal of literature exists regarding the application of growth factors in other types of wounds that could be extrapolated to the burn wound (Moura et al., 2013; Picard et al., 2015; Zarei et al., 2018).

The most common method to deliver growth factors is the topical application of growth factor solutions, creams, or gels. In a pair of seminal studies, EGF produced a dose-dependent increase in epithelialization following a burn injury in a porcine model (Brown et al., 1986). TGF-α applied at low doses led to improved healing in a PT burn model (Schultz et al., 1987). In both studies, the growth factors were mixed into an antibiotic cream and then applied topically to the wound. Topical growth factor application has also been used to augment existing treatments. For instance, a series of growth factors were applied to a burn wound covered with a skin graft in a rat model. In this study, topical delivery of keratinocyte growth factor (KGF-2), bFGF, and TGF-β_2_ improved epithelialization rates compared to skin graft alone. Interestingly, KGF-1, IL-4, and macrophage colony stimulating factor (MCSF) did not significantly improve epithelialization rates (Smith et al., 2000). A recent meta-analysis suggested that the topical administration of granulocyte-macrophage colony stimulating factor (GM-CSF), bFGF, or EGF could shorten healing time in PT burns (Zhang et al., 2016). Despite this evidence, to date only one growth factor system has been approved by the FDA for a wound healing application: topical PDGF (Regranex® Gel) for treating chronic and not burn-related wounds (Bolton, 2016). Unfortunately, there have been no reported attempts to apply the Regranex® Gel in a burn wound. However, one US-based trial was identified that is investigating the use of recombinant PDGF in thermal burns (Table 11).

**Table 11:** Clinical trials for growth factors.

**Clinical trial #**	**Clinical trial title**	**Intervention**	**Characteristics**			
			**Enrollment**	**Model**	**Allocation**	**Phase**
NCT00812513	Efficacy of R-Pdf/Gbb in healing wounds caused by third degree thermal and electrical burns	Drug: Recombinant Human PDGF	120	SGA	–	2
NCT01553708	Effect of EGF with silver sulfadiazine cream compared with silver zinc sulfadiazine cream for treatment of burn wound	Drug: EGF with silver sulfadiazine cream Drug: Silver zinc sulfadiazine cream	34	PA	R	2, 3
NCT03279549	Research on the key technology of burn wound treatment	Procedure: Routine dressing change Procedure: Limited debridement (LD) & ADM dressing Procedure: LD & Epidermal cell spraying Procedure: LD & bFGF	200	PA	R	–
NCT01843686	Using autologous platelet rich plasma (PRP) gel to treat deep 2nd and 3rd degree burns	Device: Magellan® Other: Placebo Saline Gel and Usual and SOC	42	PA	R	1
NCT01383187	Autologous platelets concentrate and autologous thrombin for the treatment of deep burn trauma	Procedure: Autograft and PRP concentrate Procedure: Autograft	30	PA	NR	2, 3
NCT02169362	Applying platelet rich plasma (PRP) gel to acute deep partial thickness thermal injuries	Device: Autologous Platelet Rich Plasma (PRP) Gel (Magellan® Bio-Bandage™)	36	SGA	R	1
NCT00858442	Platelet-rich plasma (PRP) in reconstructive surgery on children with retractable burn sequelae on extremities	Procedure: With PRP	44	PA	R	–
NCT00856934	Effect of platelet rich plasma and keratinocyte suspensions on wound healing	Biological: Autologous Platelet Rich Plasma Biological: Keratinocyte suspension Other: Standard dressings	45	PA	R	1

–*, study did not mention; ADM, Acellular Dermal Matrix; SOC, Standard of Care; STSG, Split-Thickness Skin Graft. Model: PA, Parallel Assignment (Therapy vs. SOC); SGA, Single Group Assignment. Allocation: R, Randomized; NR, Non-Randomized*.

Instead of applying a single recombinant growth factor to treat a burn, other research has considered applying a combination of growth factors derived from allogeneic or autologous sources. Plasma-based treatments of burns are one such area of intense study. Platelet-rich plasma (PRP) contains extracted plasma purified with supraphysiological concentrations of platelets. Recent studies have yielded mixed results. A rodent burn model demonstrated that PRP accelerated wound closure and resulted in less GT compared to controls for PT burns but not FT burns (Venter et al., 2016). In contrast, a recent clinical trial failed to demonstrate any statistically significant improvement in burn healing when PRP was applied as an adjunct with an autograft (Marck et al., 2016). It should be noted that different definitions of PRP exist, and that the variable results in the literature concerning PRP might be due in part to inconsistent PRP formulations across different studies (Wasterlain et al., 2012).

In addition to the existing PRP-based studies, unique formulations of plasma for burn treatments have also been proposed. For instance, plasma was recently formulated with extremely high concentrations of growth factors, including PDGF at ~50-times the standard *in vivo* concentration (Araki et al., 2012). The potential of this formulation was demonstrated after no HTS formed after treatment of a FT burn on one finger compared to scarring on an adjacent finger with PT burns that were treated conservatively (Mashiko et al., 2016). Other efforts to prepare more durable plasma-based materials using different chemical cross-linkers have been reported. In a pair of recent studies, a polyethylene glycol (PEG)-reinforced fibrin hydrogel was used to treat burn wounds in a DPT porcine model. When applied post-debridement, the PEG-fibrin gel reduced the degree of contraction compared to untreated controls (Burmeister et al., 2018a). Using a similar porcine model, the PEG-fibrin gel was also used to deliver ASCs as an adjunct to a meshed autograft and led to improved angiogenesis (Burmeister et al., 2018b).

Advanced growth factor release strategies in TE often involve the use of microparticles, nanoparticles, or hydrogels in order to carefully control growth factor release rates or improve growth factor half-life. These approaches have been recently investigated for treating burn wounds by developing a biomatrix consisting of PDGF covalently bound to fibrin. PDGF was gradually released from fibrin via enzymatic degradation. The sustained release of PDGF from this matrix improved wound healing in a porcine grafted third degree burn model (Mittermayr et al., 2016). A more complex formulation was reported in which EGF was loaded into an artificial vesicle (termed “liposome”), which was in turn encapsulated into a chitosan gel. This liposome-in-chitosan formulation increased epithelialization rate more than a chitosan gel or EGF applied alone, possibly due to improved longevity of EGF within the liposomes (Degim et al., 2011). These efforts to modulate the release of growth factors into burn wounds can be expanded to include more advanced release strategies, such as the release of multiple factors from a single material or prolonged release of factors over time.

Plasmid DNA (pDNA) is non-chromosomal circular DNA which exploits the cell machinery to produce proteins for a transient amount of time (Scholz and Wagner, 2012). To enhance dermal regeneration after burn, a number of pDNAs that encode for pro-regenerative proteins have been investigated. For instance, a porous dermal equivalent loaded with pDNA-VEGF for local sustained production of VEGF was applied to a porcine FT burn wound. The pDNA-VEGF treated wound demonstrated faster regeneration and the development of a greater number of mature blood vessels compared to control groups (Guo et al., 2011). Intradermal injection of hypoxia inducible factor (HIF-1α) plasmid vector in combination with BMSCs improved wound healing in an elderly murine burn model which was characterized by impaired wound healing due to reduced levels of endogenous HIF-1α (Du et al., 2013).

This phase of wound healing spans the gamut on FDA approvals with allografts only requiring HCT/P, most skin substitutes receiving 510 (k) approved, and cell based substitutes requiring a clinical trial supported PMA (Tables 7, 8). These new TERM strategies are quite varied in their approach from TE skin, stem cells, growth factors, exosomes, secretomes, and gene therapy. Many if not all of these strategies will require a lengthy approval process with clinical trials (Tables 9–11 for current trials). This regulatory pathway is one of the major hurdles to eventually have these therapies in the clinical setting as SOC.

## Matrix remodeling

The final stage of wound healing is matrix remodeling which continues to progress for years after injury and when aberrant, can result in scar formation and contracture. As the proliferative phase of wound healing transitions into the remodeling phase, well organized type I collagen becomes more abundant and wound tensile strength improves, The remodeling of collagen fibers ultimately leads to ~80% wound strength by roughly 6-weeks after injury (Madden and Peacock, 1968; Diegelmann and Evans, 2004). Dependent on myofibroblasts, wound contraction occurs concurrently with remodeling and aids in wound closure. Under normal conditions, fibroblasts and myofibroblasts gradually disappear from the wound by apoptosis, but dysregulation of cell death and persistence of these cells after wound closure can lead to contractures and HTS (Sarrazy et al., 2011). Wound remodeling, specifically focusing on fibroblast/myofibroblast activity, are promising areas for improving wound healing outcomes.

The mechanisms of pathological scarring during remodeling are multifactorial and include exaggerated inflammation, prolonged re-epithelialization, overabundant ECM production, augmented neovascularization, atypical ECM remodeling, and reduced apoptosis (van der Veer et al., 2009). The molecular biology of pathologic scarring is likewise complex, with vast numbers of cytokines, growth factors, and other proteins interacting (Profyris et al., 2012). Rodent models have studied fetal regenerative healing along with adult wound healing to define the critical proteins involved. Among the most important of these molecules, TGF-β_1_ and TGF-β_2_ promote scar formation while TGF-β_3_ reduces scarring (Ferguson and O'Kane, 2004). Similarly, pro-inflammatory cytokines, namely IL-6 and IL-8, promote scarring (Liechty et al., 2000a) while anti-inflammatory cytokines, most importantly IL-10, reduce scarring (Liechty et al., 2000b). As this body of research continues to expand, the key molecular pathways and potential therapeutic targets to reduce scarring will be identified.

### HTS prevention

Currently, two FDA-approved treatments for idiopathic pulmonary fibrosis show some promise in wound healing. Nintedanib is a tyrosine kinase inhibitor that reduces myofibroblast differentiation and ECM production by dermal fibroblasts in animal models (Huang et al., 2016). Pirfenidone acts through an unknown mechanism to limit TGF-β signaling (Macías-Barragán et al., 2010). In clinical studies, this small molecule outperformed compression therapy in a trial of pediatric patients with established hypertrophic burn scars (Armendariz-Borunda et al., 2012; Janka-Zires et al., 2016). These novel therapies need to be further evaluated in the burn population in order to establish their safety and efficacy before widespread adoption is feasible (Table 13).

pDNA and RNA interference (RNAi) strategies have advanced significantly over the past 30 years. Small interfering RNA (siRNA) and miRNA play a role in RNAi pathways by post-transcriptional regulation of gene expression for a period of time (Lam et al., 2015). Currently, pDNA and RNAi strategies for HS treatment are still in their infancy and are primarily being developed *in vitro* and evaluated in the rabbit HS ear model (Li et al., 2011; Wang et al., 2014; Guo et al., 2017). However, Castleberry et al. developed a layer-by-layer siRNA delivery system to target the expression of connective tissue growth factor (CTGF), a key mediator of the TGF-β1 pro-fibrotic response. In a full-thickness rat burn model, knockdown of CTGF significantly altered the expression of αSMA, tissue inhibitor of metalloprotenase-1 (TIMP-1), and type 1 collagen. The RNAi treatment resulted in improved tissue remodeling and a reducing in total scar area and contraction (Castleberry et al., 2016).

### HTS mitigation

Despite a myriad of SOC scar treatment options including surgery, compression, silicone dressings, intralesional steroid or antimetabolite injection, laser therapy, cryotherapy, and others, published or ongoing clinical research for novel hypertrophic burn scar treatments are limited and frequently include case series or only small clinical trials. These novel therapies target specific molecular pathways vital to matrix remodeling and abnormal collagen deposition.

In a Phase II clinical trial of patients undergoing scar revision surgery, the recombinant TGF-β_3_ avotermin improved scar appearance when administered immediately following surgery (So et al., 2011). While use of Integra® as a dermal replacement is well established; recently, the use of Integra® in the form of a flowable powder demonstrated efficacy by reducing post-burn scars associated with joints (shoulders, hands, and arm) and improved their range of motion (Hirche et al., 2016). Injection of adipose tissue into burn scars was shown to downregulate TGF-β levels, reduce fibroblast numbers, and halt VEGF production 6 months after treatment, leading to improved scar texture and appearance (Bruno et al., 2013). In a porcine burn study, subcutaneous injections of ASCs or fresh lipoaspirate were delivered to the HTS and reduced scar thickness was demonstrated with both the purified stromal cells and fresh adipose tissue compared to control (Rapp et al., 2017). In other pre-clinical research, topical application of a TGF-β antagonist (Singer et al., 2009) or nitric oxide (Singer et al., 2017) improved healing time and reduced scar thickness in porcine PT burns. In another, porcine scars were treated subcutaneously with recombinant human tropoelastin, ultimately increasing tissue elastin but without a demonstrable effect on scar hardness, flexibility, or inflammation (Xie et al., 2017).

While the technology is not new, lasers are currently in 11 listed clinical trials to determine their safety and efficacy for scar mitigation (Table 12). Pulsed dye lasers administer light to target hemoglobin specifically and coagulate the abundant microvasculature that supports excessive cell and ECM proliferation in scars (Hultman et al., 2012). Fractional photothermolysis with the ablative CO_2_ or the erbium-doped yttrium aluminum garnet (Er:YAG) lasers create microscopic dermal injuries that renew the healing process on a smaller scale (Tierney et al., 2009). This new cycle of healing occurs with improved collagen deposition and fibroblast apoptosis, leading to improved scar texture and appearance. To achieve suitable results, though, multiple laser treatments may be needed, each carrying associated risks including pain, blistering, and long-term skin discoloration. The available literature reports these complication rates inconsistently, and robust clinical evidence with laser therapy, such as multi-center, randomized, controlled trials, is lacking (Zuccaro et al., 2017). In an effort to combine modalities, ablative lasers may be useful in enhancing the delivery of topical steroids into the scar tissue (Waibel et al., 2013).

**Table 12:** FDA approved therapies for scar mitigation.

**Product, company**	**Indication**	**Composition**	**Format**	**FDA approval**
Integra® Dermal Regeneration Template, Integra Life Sciences	For repair of scar contractures when other therapies have failed or when donor sites for repair are not sufficient or desirable due to the physiological condition of the patient	Chemically cross-linked bovine collagen and GAGs with and without on a semipermeable silicone backing	Hydrated solid and meshed sheets stored at RT	PMA
Lumenis UltraPulse CO_2_ Laser, Lumenis, Inc.	Incision/excision and vaporization of soft tissue to include debridement of burns and laser skin resurfacing for the reduction, removal, and/or treatment of surgical scars and keloids	High energy CO_2_ laser at 10.6 μm wavelength	Causes microscopic dermal injury, leading to renewed healing	510 (k)
Vbeam Pulsed Dye Laser System, Candela Corp.	Treatment of benign cutaneous lesions such as scars	High energy 595 nm flash-lamp excited pulsed dye medical laser	Targets hemoglobin to photocoagulate skin microvasculature	510 (k)

*CO_2_, Carbon Dioxide; GAG, Glycosaminoglycan; PMA, Pre-market Approval; RT, Room Temperature*.

The area of scar prevention and mitigation is becoming more important with the increase in survivability of severely burned patients. There is a paucity of approved therapies aimed at improving the long term outcomes of this debilitating injury. Tables 12, 13 are disproportionately tilted toward laser therapies. More therapies are needed from a prevention standpoint but the issue is that not every burn scars nor does every patient. To design an appropriate RCT is difficult in these situations.

**Table 13:** Clinical trials for scar prevention and mitigation strategies.

**Clinical trial #**	**Clinical trial title**	**Intervention**	**Characteristics**			
			**Enrollment**	**Model**	**Allocation**	**Phase**
NCT03026270	Effect of therapeutical paraffin in the malleability of burned skin	Other: Paraffin	60	CA	–	–
NCT01018589	Cicatrix cream in post-surgical scars and epidermic burn	Other: Cicatrix cream	100	SGA	–	2
NCT02417818	Cutaneous microcirculation after plasma therapy	Device: Plasma Therapy (PlasmaDerm) Device: Repetitive Plasma Therapy (repetitive PlasmaDerm)	240	PA	R	–
NCT03352297	Nanofat in post-burn scars on the face	Biological: Unfiltered Nanofat Graft	48	SGA	–	–
NCT02014298	Non ablative fractional laser (NAFL) treatment of burn scars	Radiation: NAFL Other: control	20	PA	R	–
NCT01619917	The role of fractional vascular laser therapy in the management of burn scars	Device: Fractional Vascular Laser	12	SGA	R	–
NCT03197649	CO_2_ laser phototherapy for management of mature burn scars	Procedure: Laser phototherapy treatment	50	PA	R	–
NCT03416660	Efficacy of different densities of fractional carbon dioxide in treatment of post-burn scars	Device: Fractional CO_2_ laser	25	PA	R	–
NCT03240718	Pilot study of the ablative fractional CO_2_ laser in hypertrophic scars in adult burn patients	Procedure: AFCL	12	PA	R	–
NCT02115646	Fractionated carbon dioxide laser and burn scar contractures: evaluation of post-treatment scar function and appearance	Device: AFCL	36	SGA	–	–
NCT03433664	Carbon dioxide laser treatment in burn-related scarring	Device: AFCL	19	PA	R	–
NCT00969215	Burn scar appearance after treatment with fractional carbon dioxide (CO_2_) laser	Device: AFCL	20	CA	R	–
NCT02707627	Laser therapy for pediatric burn scars	Device: AFCL Procedure: Standard Scar Management Device: PDL	54	PA	R	–
NCT00852280	Effects of pulsed-dyed laser on scar formation	Device: Treatment of 1/2 of skin graft with PDL	17	SGA	R	–
NCT01488240	The role of pulsed dye laser therapy in the management of burn scars	Procedure: PDL	12	PA	R	–

–*, study did not mention; AFCL, Ablative Fractional CO_2_ Laser; CO_2_, Carbon Dioxide; PDL, Pulsed Dye Laser; STSG, Split-Thickness Skin Graft. Model: CA, Crossover Assignment; Ob, Observational; PA, Parallel Assignment (Therapy vs. SOC); SGA, Single Group Assignment. Allocation: R, Randomized; NR, Non-Randomized*.

## Discussion

The current accepted practice for the treatment of burn wounds is early excision of the necrotic tissue followed by immediate autografting. This strategy reduces microbial colonization, improves survival, shortens hospital stay, and decreases HTS formation (Sterling and Heimbach, 2011). In the event of a large TBSA injury or in an attempt to reduce autograft requirements, a variety of epidermal and dermal products have been discussed in this article that may facilitate wound closure. Proper wound bed preparation including adequate debridement, hemostasis, and infection control is paramount prior to autografting in order to prevent graft failure; this is also true with the skin substitutes. The depth of burn and presence of dermis dictates which products can be used alone or in combination, such as a dermal substitute followed by an epidermal substitute. In addition to minimizing the use of donor skin, the research and development of TERM products aims to mitigate the incidence of infection, provide temporary and/or permanent coverage, and accelerate re-epithelialization during the acute stages of wound healing. What is still missing is comprehensive RCT of these products comparing efficacy to each other. One simple example would be a head-to-head comparison of all silver dressings but this can be extrapolated to other topics discussed in this review (i.e., dermal matrices). Exploiting the synergy of combined attributes is where TERM can provide the greatest benefit, such as the development of an antimicrobial full-thickness skin substitute. Moving forward, one of the greatest challenges for the use of TERM products is in deciding which therapies are compatible and also have complementary functions while successfully fitting into the FDA requirements. Definitive RCTs will answer these questions and determine the safety and efficacy of combinatorial approaches. With an understandable emphasis on acute care in the literature, current clinical gaps need to address the inflammatory response, as well as the proliferative and remodeling phases of wound healing. Stem cell secretome and other biological-based therapeutics working as an adjunct to skin substitutes can guide cell proliferation to enhance TERM product integration while potentially preventing scarring. Another major challenge is navigating the FDA approval process successfully. Novel TERM approaches that cannot rely on a “predicate” device to receive 510 (k) approval must undergo clinical trials prior to approval which is costly in terms of actual cost but also time. This inherently limits the advancement of new ideas and technologies that could significantly improve the functional and cosmetic outcomes of burn victims. Although TERM strategies have expanded our understanding of skin biology and physiology, no therapy is currently available that truly replaces skin, restores function (e.g., pigmentation, hair follicles, glands, elasticity, and nerves), and prevents scarring.

## Author contributions

RS: wound healing cascade, hemostasis section, discussion, compilation of article, tables; SN: burn wound coverings and stem cells, figures; CK: infection, gene therapy, tables; LM: immune modulation; RMC: burn wound coverings; NC: growth factors; AC, DT, and RKC: scar prevention and mitigation; JR: current standard of care and clinical gaps; RJC: participated in preparation and revision of manuscript. All authors approved the final version of the manuscript.

### Conflict of interest statement

The authors declare that the research was conducted in the absence of any commercial or financial relationships that could be construed as a potential conflict of interest.

## Abbreviations

α-SMA: alpha smooth muscle actin
AM: amniotic membrane
AMP: Antimicrobial peptides
ASC: adipose tissue derived mesenchymal stem cells
AATB: American Association of Tissue Banks
bFGF: basic fibroblast growth factor
BLA: Biological License Application
BMSC: bone marrow derived mesenchymal stem cells
CEA: cultured epithelial autograft
COX-2: cyclooxygenase-2
CSS: cultured skin substitutes
CXCL2: chemokine (C-X-C motif) ligand 2
DCs: dendritic cells
DPT: deep partial-thickness
ECM: extracellular matrix
EGF: epidermal growth factor
ESS: engineered skin substitutes
FDA: Food and Drug Administration
FLT-3: fms-like tyrosine kinase-3 ligand
FT: full-thickness
GM-CSF: granulocyte-macrophage colony stimulating factor
GT: Granulation Tissue
HCT/Ps, Human cells, tissues: and Cellular and Tissue-based Products
HDE: Humanitarian Device Exemption
HFSC: hair follicle stem cells
HIF: hypoxia inducible factor
HTS: Hypertrophic scars
IDE: Investigational Device Exemption
IL: interleukin
IFN-γ: interferon gamma
IND: Investigational New Drug
KGF: keratinocyte growth factor
MCSF: macrophage colony stimulating factor
miRNA: microRNA
MMP: matrix metalloproteinase
MRSA: Methicillin-resistant *Staphylococcus aureus*
MSC: mesenchymal stem cells
NDA: New Drug Application
NO: nitric oxide
NSAIDS: non-steroidal anti-inflammatory drugs
PDGF: platelet derived growth factor
pDNA: Plasmid DNA
PGE_2_: Prostaglandin E_2_
PMA: Premarket Approval
PMNs: polymorphonuclear leukocytes or neutrophils
PRP: platelet rich plasma
PT: partial-thickness
RCT: randomized control trials
RNAi: RNA interference
SDF: stromal derived factor
siRNA: Small interfering RNA
SOC: standard of care
SPT: superficial partial-thickness
SSD: silver sulfadiazine
SVF: stromal vascular fraction
TBSA: total body surface area
TE: tissue engineering
TERM: tissue engineering and regenerative medicine
TGF-β: transforming growth factor beta
TIMP: tissue inhibitor of matrix metalloproteinase
TNF-α: tumor necrosis factor alpha
VEGF: vascular endothelial growth factor.
